# Use of matrix-assisted laser desorption/ionization mass spectrometry imaging (MALDI-MSI) to visualize and support interpretation of toxic effects of 4-hydroxyphenylpyruvate dioxygenase inhibitors in rat tissues

**DOI:** 10.1007/s00204-025-04175-0

**Published:** 2025-09-30

**Authors:** R. Schneider, M. Giampà, M. C. Schröder, M. Kubicki, J. Boyken, L. Beuret, G. Semino-Beninel, K. Niehaus, F. Schorsch, M. Lamshoeft, H. Bednarz

**Affiliations:** 1 Proteome and Metabolome Research, Center for Biotechnology (CeBiTec), Faculty of Biology, Bielefeld University, Bielefeld, Germany; 2 Division Crop Science, Bayer AG, Monheim, Germany; 3 Division Crop Science, Bayer S.A.S, Sophia Antipolis, Valbonne, France; 4 Division Pharmaceuticals, Bayer AG, Berlin, Germany; 5 Medical School OWL, Anatomy and Cell Biology, Bielefeld University, Bielefeld, Germany; 6 Center for Cancer Biology, VIB-KU Leuven, Leuven, Belgium

**Keywords:** HPPD inhibitor, Tyrosine, Metabolomics, Mass spectrometry imaging, Iodide accumulation

## Abstract

4-Hydroxyphenylpyruvate dioxygenase inhibitors (HPPDi) are mainly used as herbicides and for therapeutic use in genetic diseases of tyrosine catabolism. Their primary mechanism of action is the inhibition of the second enzyme of tyrosine catabolism, leading to an accumulation of this amino acid in blood and tissues. In this work, rats were administered diets with 1 ppm, 2 ppm, and 10 ppm of HPPD-inhibitor BCS-CR75391 for up to 28 days, and tyrosine levels were measured in blood and in selected organs using mass spectrometry combined with gas or liquid chromatography and analyzed spatially using mass spectrometry imaging (MSI). The highest tyrosine accumulation was recorded in the pancreas, followed by the eyes and the thyroid gland. Metabolomic profiling showed that other amino acids and metabolites of the citric acid cycle were influenced by the treatment. A metabolic adaptation was observed in the liver and kidney 28 days after the treatment, but not in other tissues analyzed. MSI of the thyroid gland seems to reveal an uneven accumulation of tyrosine in the tissue of rats following treatment with BCS-CR75391. Most interestingly, a significant accumulation of iodide was detected in the thyroid gland of all rats treated with the 4-hydroxyphenylpyruvate dioxygenase inhibitor. In addition, induced high tyrosine levels by a tyrosine-rich diet also provoke the accumulation of iodide in the thyroid gland. While the toxicological impact of these results needs to be further investigated, these results support the use of MSI as an innovative and powerful tool to support toxicological assessment.

**Supplementary Information:**

The online version contains supplementary material available at 10.1007/s00204-025-04175-0.

## Introduction

The 4-hydroxyphenylpyruvate dioxygenase inhibitors (HPPDi) are a chemically heterogeneous class of active agents that affect the catabolic pathway of tyrosine and are mainly used as herbicides, but interestingly also as drugs for the treatment of tyrosinemia type I and alkaptonuria (Antonenko et al. 2015; Lock 2017). Most of the developed HPPDi belong to the chemical class of triketones and benzoyl pyrazoles (Antonenko et al. 2015). The main mechanism of action of the HPPDi is the inhibition of 4-hydroxyphenylpyruvate dioxygenase (HPPD), a non-heme Fe (II)-oxidase of the catabolic tyrosine pathway that catalyzes the conversion of 4-hydroxyphenylpyruvate (HPPA) into homogentisic acid and carbon dioxide (Lewis and Botham 2013). The reason why HPPDi are excellent candidates for herbicides is because they induce the depletion of homogentisic acid. For plants, homogentisic acid is a necessary precursor for the biosynthesis of α-tocopherol and plastoquinone (Almsick et al. 2013). As plastoquinone plays a role in carotenoid biosynthesis and α-tocopherol has an antioxidant function itself, this path is an important part of the protection against oxidative stress for the plants (Almsick et al. 2013). Triggered by these processes, the so-called bleaching effect ultimately occurs due to the destruction of the chlorophyll and plant growth is inhibited until it dies off (Almsick et al. 2013). In mammals, the inhibition of HPPD primarily leads to an accumulation of tyrosine in the blood, the so-called tyrosinemia (Ellis et al. 1995; Lewis and Botham 2013; Antonenko et al. 2015). However, the onset of tyrosinemia and tyrosinemia-associated toxicological effects following exposure to HPPDi are different across mammalian species. This is because when HPPD is inhibited, the clearance of excess tyrosine is driven by tyrosine aminotransferase (TAT), and it has been demonstrated that the activity of this enzyme varies between species. Species with higher TAT activity (like human and mice) show lower systemic tyrosinemia while others (like dogs and rats) with lower TAT activity show higher tyrosinemia and toxic effects. Therefore, the rat is the most susceptible species to the toxic effects of tyrosinemia induced by an HPPDi (Botham et al. 2023). In this species, tyrosine accumulation was also observed in the aqueous humor of the eye, resulting in corneal irritation and opacity of the cornea (Lock et al. 1996). Other observations were body weight decrease, liver and kidney weight increase, as well as morphological changes in the exocrine pancreas and in the thyroid gland (Lewis and Botham 2013). In this latter, colloid alteration has been sometimes reported, but there has so far been no mechanistic data that could indicate a correlation between those effects and tyrosinemia.

Various well-established mass spectrometric methods, combined with chromatographic separation steps such as liquid chromatography (LC) or gas chromatography (GC), are particularly suitable for metabolic analyses of complex samples (van Ravenzwaay et al. 2007). Using these techniques, extracts from any organ tissue can be examined in the best possible way to identify and quantify the metabolites they contain. However, these techniques are not able to provide information concerning the native localization of these metabolites within an organ or a tissue. In Mass Spectrometry Imaging approaches, such as Matrix-Assisted Laser Desorption/Ionization (MALDI)-MSI the localization of metabolites of interest can be accurately determined by direct measurements in tissue sections (Bednarz and Niehaus 2021). For this reason, MSI is the so far missing link between histology and physiology.

The main objective of the present study is to provide information on time-dependent and dosage-dependent metabolic alterations following administration of an HPPDi in the rats, to quantify the accumulation of tyrosine, to profile metabolic changes, and to visualize distributions of tyrosine and other metabolites in organs, which has been reported as being sensitive to tyrosinemia.

## Methods

### Animal study, HPPDi and tyrosine administration and sample collection

Approximately 7-week-old male Wistar Crl:WI Han rats were used for the animal experiments (Charles River Laboratories, France). The animals were always kept in a defined environment of a 12 h day–night cycle (12 h light/12 h dark). The light source was automatically controlled fluorescent tube lighting. The temperature during the experiment was 20–24 °C, and the relative humidity between 40 and 70%. Sufficient and continuous fresh air supply was ensured (10 to 15 air changes per hour). The animals had access to water and food ad libitum. Filtered and softened tap water from the municipal water supply was used for this purpose. The food used was the certified powdered and irradiated rodent diet A04CP1-10 with 16% protein and 0.44% tyrosine from Scientific Animal Food and Engineering, France.

The animal study was performed according to standard operating procedures which have been previously accepted and periodically inspected by the quality assurance unit. Before the animals were exposed to BCS-CR75391 (a potent HPPDi which was not further developed), a nine-day acclimatization period was conducted. The test items were administered orally. Either the appropriate dose of HPPDi BCS-CR75391 (purity: 98.4% w/w) was added to the diet, or the diet contained an increased amount of L-tyrosine of up to 5% of the total content.

Dose groups with 1 ppm, 2 ppm, and/or 10 ppm BCS-CR75391 were established and compared to a control group. Two dose groups with 1 ppm and 10 ppm were established and compared to a control group over 14 days of exposure. In addition, a dose group treated with 2 ppm BCS-CR75391 was compared to  a group which received a 5% w/w tyrosine-enriched diet and the control group over 28 days of exposure. The concentrations were selected based on the results of preliminary studies showing that these doses induced severe tyrosinemia in rats. The feed was available to the animals for 14 and 28 days. On day 15 and 29, the animals were sacrificed. This was followed by necropsy (Table 1).

**Table 1:** Summary of treatments

Studies	14 days	28 days
Control diet	Yes	yes
BCS – CR75391	1 ppm	
		2 ppm
	10 ppm	
Tyrosine enriched died	no	5% w/w

Sacrifice was performed in an anesthetized state. The animals were anesthetized with the help of isoflurane and then exsanguinated in the state of deep anesthesia before necropsy. During necropsy, the eyes, thyroid glands, livers, kidneys, and pancreas of each animal were collected. The organ samples were treated in two different ways. First, they were snap frozen in liquid nitrogen. For this, the right eyes, the right half of the thyroid, the right kidney, and two pieces of the pancreas and two pieces of the median and left lobe of the liver were used. After snap freezing in liquid nitrogen, the samples were stored at − 80 °C until further use. The other half of the samples was preserved in 10% neutral buffered formalin. For this purpose, the left parts of the thyroid glands, the left kidneys, a part of the pancreas, and the left lobes of the livers were used. The respective left eyes were preserved in Davison fixative. Fixation was followed by embedding in kerosene wax. The fresh frozen (FF) tissue samples were used for gas chromatography-mass spectrometry (GC–MS), liquid chromatography–mass spectrometry (LC–MS), MALDI-MSI, and laser ablation-inductively coupled plasma-mass spectrometry (LA-ICP-MS), while the formalin-fixed paraffin-embedded (FFPE) samples were used mainly for histopathological examination but also for MALDI-MSI (Table 2).

**Table 2:** Overview on samples, sample preparation and analysis carried out in this study

Sample type	Preparation	Analysis	Exposition period	Number of samples per group	Number of animals per group
Blood	Mixed with 7% perchloric acid storage at − 20 °C	HPLC (L-tyrosine)	14 days	14	14
Thyroid gland	Snapfrozen (liquid nitrogen) Storage at − 80 °C	UHPLC-MS (amino acids, L-tyrosine)	14 days	12 (control group = 24)	12 (control group = 24)
		MALDI-MSI (L-tyrosine + iodide localization)	14 days	1	1
			28 days	L-tyrosine = 6/iodide = 4	L-tyrosine = 4/iodide = 4
		LA-ICP-MS (iodide localization)	28 days	3	2
Thyroid gland (FFPE)	Formalin-fixed (10% neutral buffered formalin) and paraffin-embedded Storage at 4 °C	MALDI-MSI (iodide localization)	28 days	4	4
Pancreas	Snapfrozen (liquid nitrogen)	UHPLC-MS (amino acids, L-tyrosine)	14 days	12 (control group = 24)	12 (control group = 24)
	Storage at − 80°C	GC-MS (metabolite profiling)	14 days 28 days	20	10
Eye	Snapfrozen (liquid nitrogen)	UHPLC-MS (amino acids, L-tyrosine)	14 days	12 (control group = 24)	12 (control group = 24)
	Storage at − 80°C	GC-MS (metabolite profiling)	14 days 28 days	20	10
Liver	snapfrozen (liquid nitrogen)	UHPLC-MS (amino acids, L-tyrosine)	14 days	12 (control group = 24)	12 (control group = 24)
	storage at − 80°C	GC-MS (metabolite profiling)	14 days 28 days	20	10
Kidney	snapfrozen (liquid nitrogen)	UHPLC-MS (amino acids, L-tyrosine)	14 days	12 (control group = 24)	12 (control group = 24)
	storage at − 80°C	GC-MS (metabolite profiling)	14 days 28 days	20	10

### Ultra-high performance liquid chromatography-mass spectrometry (UHPLC-MS) sample preparation and analysis

Quantification of tyrosine was performed after extraction from tissues by means of UHPLC-MS. The pretreatment was performed with the EZ:faast Amino Acid Analysis Kit (Phenomenex Inc., Torrance, CA, USA). 1 ml of a methanolic extract (70% methanol) from 10 mg dry weight of the respective organ material was used as starting material for the kit. These analyses are based on a selective purification of amino acids and amine compounds via solid phase extraction (SPE), steps of concentration, and a derivatization with propyl chloroformate. Measurements were performed using a Dionex Ultimate 3000 (Thermo Fisher Scientific, Waltham, MA, USA) coupled to the QTOF mass spectrometer micrOTOF_Q_ (Bruker Daltonics GmbH & Co. KG, Bremen, Germany). The column was a modified C18 with a diameter of the column material of 4 µm (AAA-MS 250 × 2.0 mm) which is part of the EZ:faast Amino Acid Analysis Kit.

The measurements were performed with the two eluents: 10 mM ammonium formate in H_2_O and 10 mM ammonium formate in methanol. A volume of 2 µl per sample was injected. All other parameters, such as elution gradient, column temperature, etc., can be found in the manual of the analysis kit.

### GC–MS sample preparation and analysis for non-target profiling of metabolites

GC–MS was chosen for non-targeted metabolite profiling because it offers excellent separation performance and reproducibility for small-molecule analytes that play a central role in basic metabolic pathways. 7 mg (± 0.1 mg) of tissue material from lyophilized kidneys, pancreas, livers, and eyes was extracted in 1 ml of ice-cold methanol/H_2_O (80:20, v/v), with 10 µM ribitol as internal standard. 0.5 g of zirconia/silica beads (0.5 mm) was added to each vial, and the mixtures were processed in a homogenizer (Precellys 24 Homogenisator, Bertin Technologies, Montigny-le-Bretonneux, France) three times for 60 s at 6200 rpm, with 15 s of break in between. The samples were subsequently centrifuged for 20 min at 14,000 rpm, and 750 µl of the respective clear supernatant was dried in a nitrogen flow at 37 °C. The automatic derivatization and measurements were performed using a Trace™ 1310 gas chromatography system coupled to the triple Quadrupole mass spectrometer TSQ 9000 (Thermo Fisher Scientific Inc., Waltham, MA, USA). Derivatization was performed with methoxylamine hydrochloride (MeOX; Merck, Sigma Aldrich) and N-methyl-N-(trimethylsilyl)trifluoroacetamide (MSTFA; Macherey–Nagel, Düren, Germany). Here, 75 µl of 20 mg/ml MeOX reagent in pyridine was added to the sample aliquot. After 1.5 h of shaking at 37 °C, 75 µl of MSTFA was added. Shaking took place again for 30 min at 37 °C, and 1 μl of the derivatized samples was injected. An OPTIMA 5 MS column (Macherey–Nagel, Düren, Germany) was used for the chromatographic separation (DB-5 MS capillary column (0.25 mm inner diameter, 0.25 μm film thickness) with 95% methyl and 5% phenyl groups). A temperature gradient method was used with an initial temperature of 80 °C that was held for 3 min, and a ramp of 5 °C/min up to 320 °C. Afterwards, the temperature was decreased by 50 °C/min to the initial temperature of 80 °C with a dwell time of 5 min. The fluid phase was helium gas with a flow rate of 1 ml/min. Mass spectrometry was performed in the range of 50 to 500 m/z.

### Thyroid tissue preparation and histological HE-staining

Both FF and FFPE tissue samples from the thyroid glands of the investigated rats were used for MALDI-MSI, LA-ICP-MS, and histological examination in this study. FF thyroid tissue samples were cut into 10 µm thick sections using a cryostat (Leica CM1850; Leica Biosystems, Wetzlar, Germany) and mounted onto an indium tin oxide (ITO) coated glass slide (Bruker Daltonics GmbH & Co. KG, Bremen, Germany). Afterwards, samples were dried for at least 30 min in a vacuum desiccator. Subsequent sections were mounted on a glass slide (HistoBond®, Paul-Marienfeld GmbH, Lauda, Germany) for histological staining. The FFPE thyroid samples were cut into 10 µm thick sections using a sledge microtome. These were placed on warm water immediately after sectioning so that they could spread and a proper straight tissue section could be mounted on the slide. Afterwards, the slides with the tissue sections were placed on a section extender (Gerhardt Analytical Systems, Königswinter, Germany) for another 20 min (45 °C).

Finally, prior to the matrix application, the deparaffinization took place. In this procedure, the paraffin is removed from the tissue sections using xylene (2 × 8 min) (≥ 98%; Sigma Aldrich, St. Louis, MO, USA). Subsequently, the sections were dried for at least 10 min in a vacuum desiccator. The slides for mass spectrometry imaging were stored in a vacuum desiccator until further use, whereas those for histology were stored in a dry cabinet. The matrices used for MALDI-MSI analyses were both 1,5-diaminonaphthalene (DAN) (97%; Sigma Aldrich, St. Louis, MO, USA) as a 10 mg/ml solution in 70% acetonitrile (HPLC Grade; VWR International LLC, Radnov, PA, USA) for FF samples and N-(1-naphthyl)ethylenediamine dihydrochloride (NEDC) (≥ 99%; Carl Roth, Karlsruhe, Germany) as a 7 mg/ml solution in 70% methanol (HPLC Grade; Thermo Fisher Scientific, Waltham, MA, USA) for FFPE samples.

Matrix application was performed using the TM sprayer (HTX-Technologies, LLC, New York, USA) and following parameters: 70 °C, 10 psi, 0.1 ml min^−1^, 1200 mm min^−1^, 14 passes in a Criss-Cross pattern with a 3 mm spacing for the matrix DAN and the parameters: 70 °C, 10 psi, 0.12 ml min^−1^, 1200 mm min^−1^, 28 passes in a Criss-Cross pattern with a 3-mm spacing for the matrix NEDC. The samples were analyzed directly afterwards or stored in a dry cabinet until measurement (max. 48 h). Subsequent tissue sections were stained with hematoxylin and eosin (HE) (Meier’s acidic hemalaun and aqueous eosin G solution (5%), Carl Roth, Karlsruhe, Germany). All staining solutions were filtered directly before use. For HE staining of the tissue sections, they were first fixed in pure methanol (HPLC Grade; Thermo Fisher Scientific, Waltham, MA, USA) (2 min). A washing step (10 dips) in demineralized water (dH2O) followed. The acidophilic structures of the tissue were then stained with the hematoxylinhemalaun solution (6 min). This was followed by another washing step in dH2O (10 dips). Afterwards, the section was blued under running tap water (10 min). Subsequent counterstaining of the basophilic/eosinophilic structures was realized using Eosin G (8 s), after a short washing step with dH2O. Further washing and differentiation steps were performed with fresh 100% ethanol (technical) (2 × 10 dips). The slides were treated with xylene (≥ 98%; Sigma Aldrich, St. Louis, MO, USA) and conserved with a synthetic mounting medium and a cover slip.

### MALDI-Orbitrap-MSI analyses

The MALDI-Orbitrap-MSI analyses were conducted in terms of targeted analysis and visualization of the distribution of tyrosine within the tissue samples of animals exposed to HPPDi, animals that were fed with a high tyrosine content diet and control animals. The measurements were performed using a Q-Exactive Plus (Thermo Fisher Scientific, Waltham, MA, USA) mass spectrometer combined with a MALDI-/ESI-Source the Spectroglyph MALDI-/ESI-Injector (Spectroglyph LLC, Kennewick, WA, USA). Details of the measurement parameters are given in the supplemental information (Supplement Table. 4—6).

### LA-ICP-MS analyses

The spatial iodide distribution was analyzed using an ICP-MS iCAP RQ (Thermo Fisher Scientific, Waltham, MA, USA) coupled to a laser ablation system (NWRimageBio; Elemental Scientific Lasers, Omaha, USA). Thin sections of thyroids were analyzed with a laser frequency of 20 Hz and a scan speed of 50 µm per second. Data processing was performed using MassImager 3.17 software, which was developed by Robin Schmid from the group of Uwe Karst (University of Muenster, Germany).

### Data processing and analysis

The HE stained tissue slides were digitalized for analysis using the Mirax Desk tissue scanner (Carl Zeiss AG, Oberkochen, Germany) and CaseViewer 2.4 software (3DHISTECH Ltd., Budapest, Hungary). Fundamental analysis of LC–MS data was performed using DataAnalysis software (Vers.: 4.0) (Bruker Corp., Billerica, MA, USA). Identification and annotation of peaks was performed according to the instructions of the used analysis kit EZ:Faast® Amino Acid Analysis Kit (Phenomenex Ltd., Torrance, CA, USA) using appropriate amino acid standards. Quantitative data analysis was performed using QuantAnalysis software (Vers.: 2.0) (Bruker Corp., Billerica, MA, USA).

Chromatographic data were processed using Xcalibur 4.2 software (Thermo Fisher Scientific, Waltham, MA, USA) and Microsoft Excel (Microsoft Corp., Redmond, WA, USA). Peak assignment and identification of metabolites were performed using the analysis of appropriate reference substances. Quantitative data were normalized as peak area ratios and statistically analyzed. A multivariate statistical analysis called principal component analysis (PCA) was performed using R (Version: 4.1.2) (R Core Team 2020) to identify differences between groups and to identify metabolic changes.

Spatially resolved MALDI-MSI data were analyzed using SCiLS Lab 2021c Pro software (Bruker Daltonics GmbH & Co. KG, Bremen, Germany), METLIN™ (Scripps Research, La Jolla, CA, USA) and the Human Metabolome Database (HMDB) 4.0 (Wishart et al. 2007).

## Results

### HPPDi treatment increased tyrosine accumulation within rat organs

**Fig. 1:**
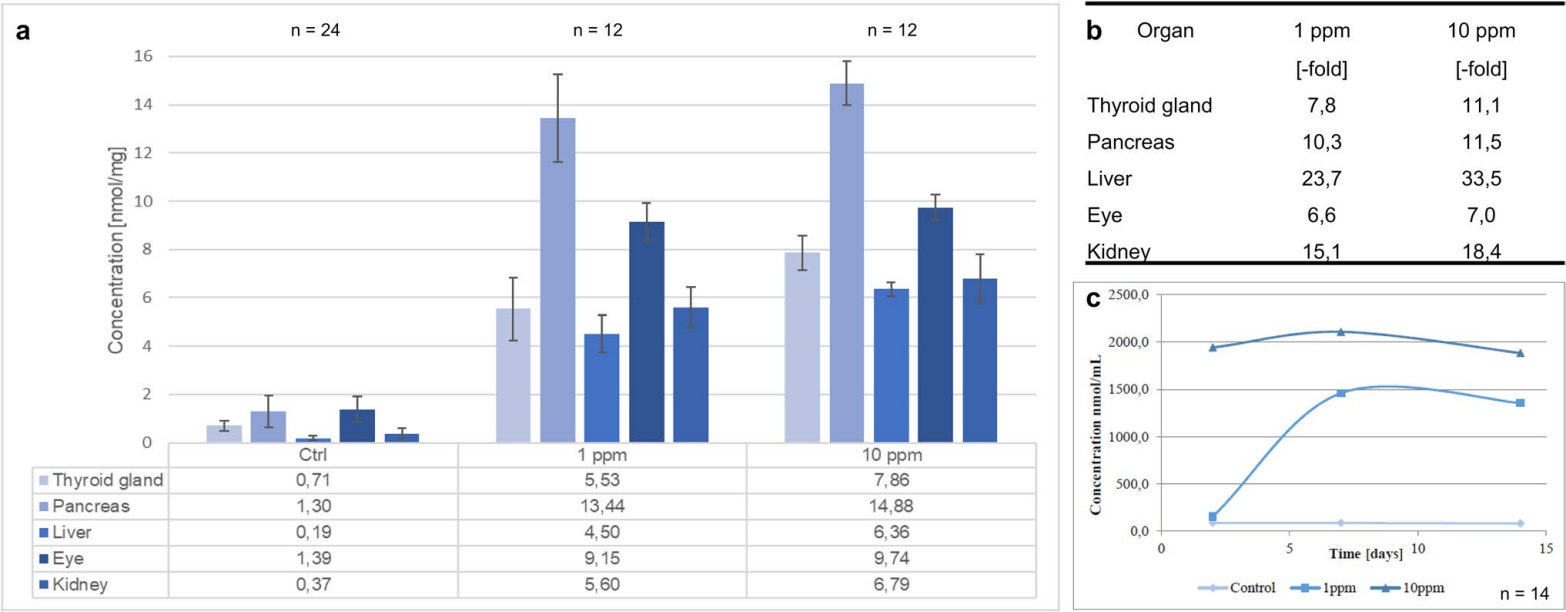
Quantitation of tyrosine within blood and tissue samples of the thyroid gland, pancreas, eye, kidney, and liver of HPPDi (BCS-CR75391) treated and control rats. Quantification of the metabolite tyrosine by UHPLC-MS within tissue samples of the rats visualized as content bar graphs **a**, the fold-change **b** of the treated groups 1 ppm and 10 ppm BCS-CR75391 in comparison to the control group, and the blood tyrosine concentrations of the examined animals **c**. HPPDi treatment for 14 days. A two-sided t-test confirms the significance of tyrosine accumulation in the blood samples of the treated animals of both dose groups compared to the controls (*p* < 0.01). When comparing the 10 ppm group with the 1 ppm group, there was a significant difference in tyrosine content only in the liver and thyroid gland (p < 0.01)

This study was planned to profile the metabolic and histopathological effects of HPPDi administration. Rats were treated with 1 ppm and 10 ppm BCS-CR75391 over 14 days. At the end of the treatment period, the amino acid concentrations within the thyroid gland, pancreas, liver, eye, and kidneys of the animals were quantified by UPLC-MS. The method used in this study was capable of the detection of all proteinogenic amino acids. The most noticeably altered amino acid was tyrosine. Tyrosine accumulated during the treatment with HPPDi not only in the blood but also in all of the examined organs. However, tyrosine accumulated at different levels depending on the tissue type (Fig. 1a, b). Quantification of tyrosine in blood samples showed that the concentration reached a plateau at the time of sampling (Fig. 1c). Alterations in tissue content in association with the administration of HPPDi were observed only for tyrosine and less evident for glutamine (see Supplement Fig. 1). Concerning all other amino acids, no changes in the tissues were detectable after the 14-day exposure, neither at a dose of 1 ppm nor at a dose of 10 ppm of HPPDi. The highest tyrosine accumulation was recorded in the pancreas, followed by the eyes and the thyroid gland. Interestingly, it can be noted that the tyrosine content of the two dose groups hardly differs, despite the tenfold dose increase. This indicates that already 1 ppm BCS-CR75391 induces a nearly saturated effect of the HPPDi. The HPPDi itself could not be detected in the pancreas or in the eye, since concentrations were under the limit of detection (< LOD). HPPDi traces were observed in the kidney, as the central excretory organ, as well as in the liver as the central site of detoxification (Supplement Table. 2 + 2). The treatment period and the administered dose have almost no effect on the amount of HPPDi that could be detected in the liver (Supplement Table. 2 + 2), indicating a rapid turnover of HPPDi in the test animals.

### Metabolic profiling via GC–MS reveals an appearance of metabolic alterations caused by 14-day treatment with HPPDi and a tendency to adaptation after 28 days of treatment

**Fig. 2:**
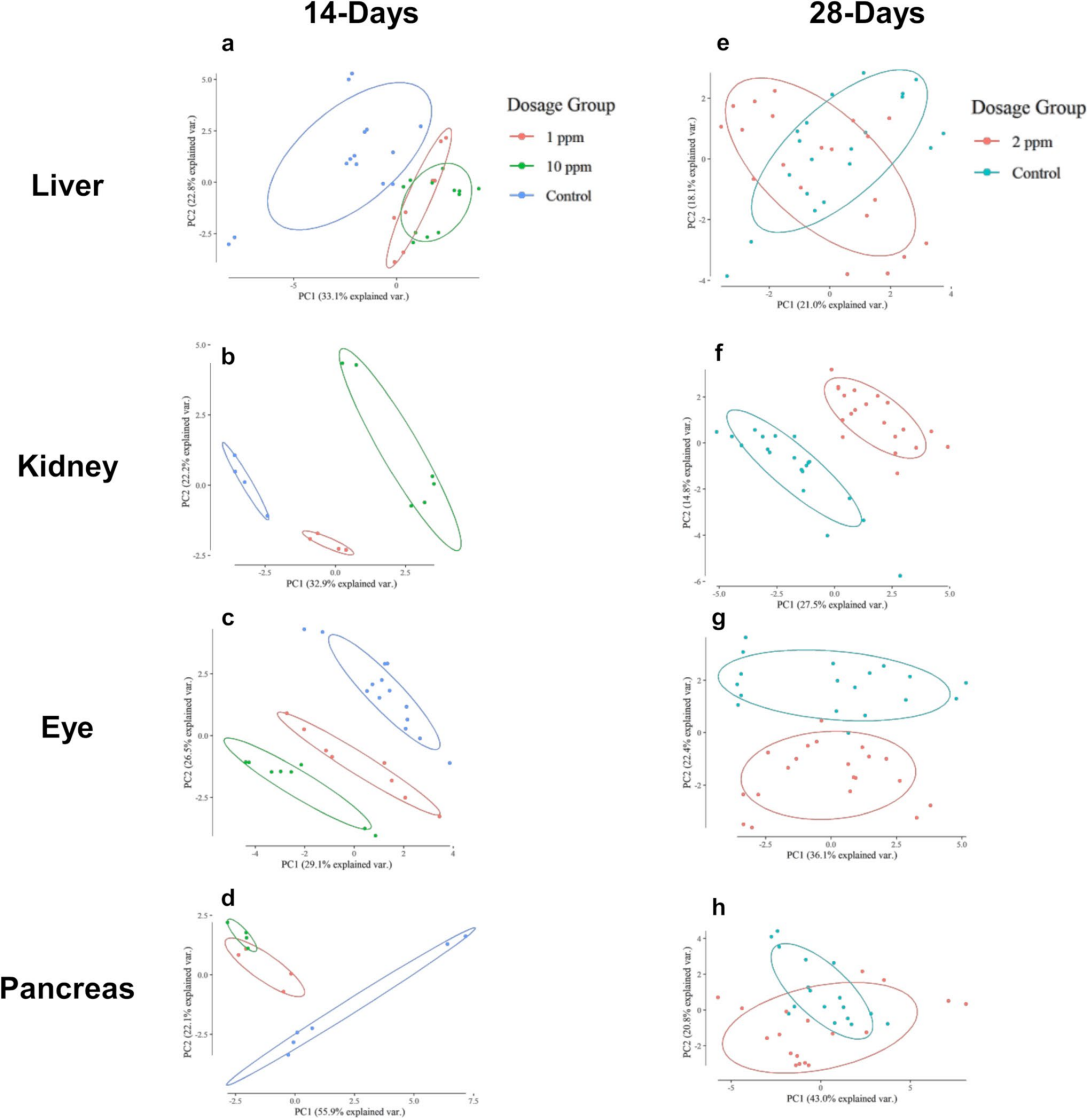
Multivariant statistics of metabolite profiles obtained by gas chromatography— mass spectrometry of organs taken from rats treated with 1 ppm, 2 ppm, or 10 ppm HPPDi for 14 or 28 days. Metabolite profiling data are presented in summary by principal component analysis (PCA) of the two dose groups receiving 1 ppm and 10 ppm of HPPDi (BCS-CR75391) over 14 days versus controls (a—d) and the animals treated with 2 ppm HPPDi for 28 days compared to controls (e—h)

Although treatment of the animals with 1 ppm and 10 ppm of BCS-CR75391 resulted in a significant increase in tyrosine levels in all analyzed organs and blood after 14 days, no morphological tissue abnormalities were observed in the eyes, thyroid gland, kidney, pancreas, or liver. Using GC–MS, the more extensive metabolic changes and a longer-term adaptation to 2 ppm HPPDi were noticed. In addition to amino acid metabolism, other fundamental metabolic pathways such as TCA cycle, glycolysis, and urea cycle could be covered by this approach. For the untargeted analysis of the GC–MS data, dimension reduction in the form of principal component analysis (PCA) was performed (Fig. 2). The PCAs of metabolic profiles from all organs of the treated animals showed a clear separation between samples from treated and control animals. Kidney and eye samples showed, in addition, a clear separation of the 1 ppm treatment versus the 10-ppm treatment in the PCA.

Only in the liver and pancreas samples was there no significant separation between these two treatment groups, although a clear separation from the non-treated controls can be observed. In the samples of the animals treated for 28 days, a change in metabolism can no longer be clearly observed in liver and pancreas tissues. The data groups (dose groups) converge and partly show a strong overlap. With longer treatment time, metabolic profiles of the tissues become more similar in these organs, indicating metabolic adaptation. After 28 days, the kidney and eye samples keep a visible separation from their respective controls, which indicates that the metabolism of these organs is still specifically affected. At this time point, characteristic lesions can be identified in the thyroid gland, the pancreas, and in the eye (Supplement Fig. 2  + 3). HPPDi induces metabolic changes in the analyzed tissues that go beyond the accumulation of tyrosine. Most metabolites showed no significant up- or down-regulation, but phenylalanine, aspartate, urea, and some metabolites of the tricarboxylic acid (TCA) cycle showed lower abundance in treated animals compared to the control (see Supplement Table 3).

### In-situ tyrosine localization by MALDI-MSI within thyroid glands of HPPDi treated rats

**Fig. 3:**
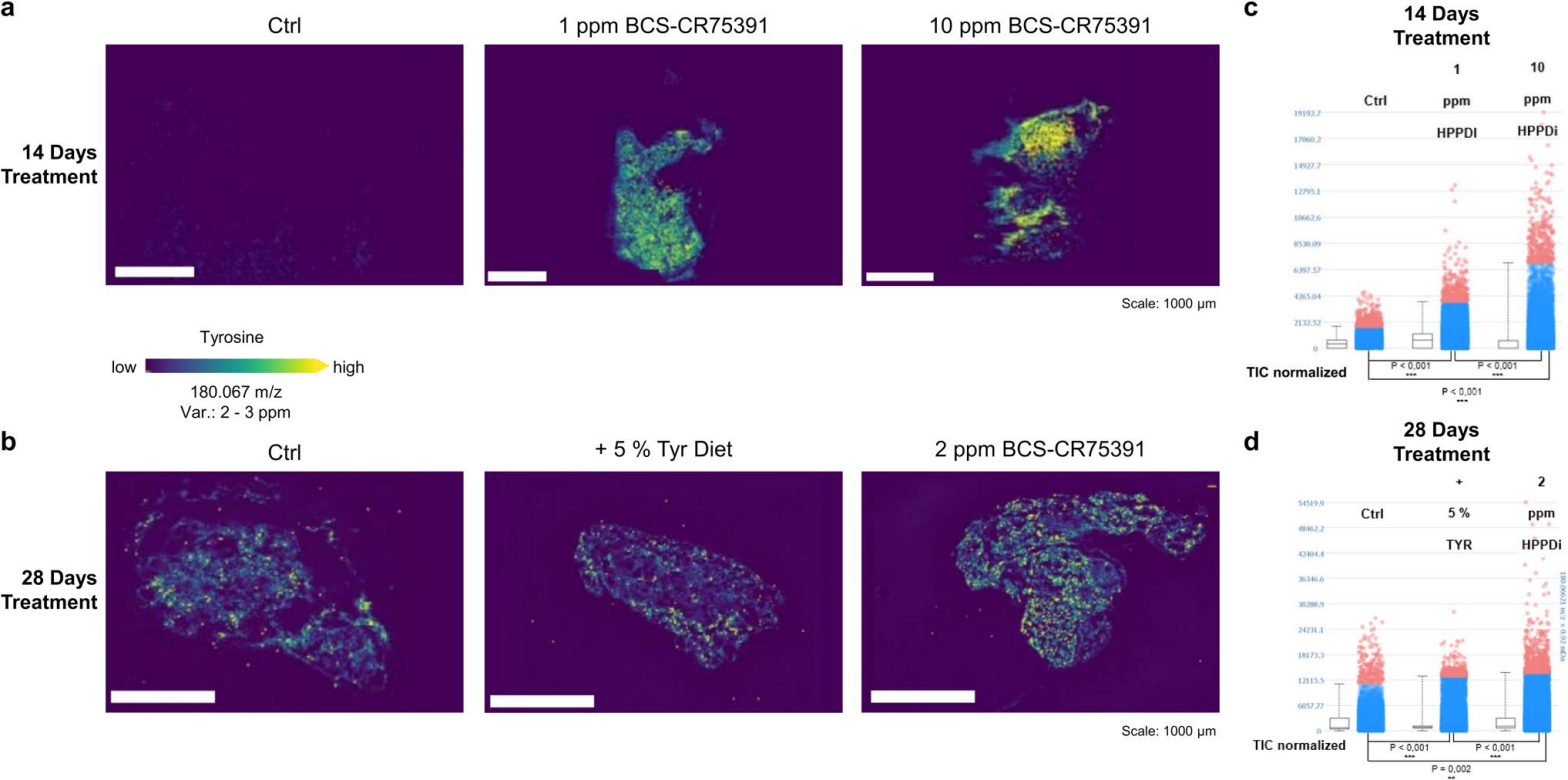
Tyrosine distribution within the thyroid gland of rats treated with 1 ppm and 10 ppm of the HPPDi BCS-CR75391 for 14 and with 2 ppm or with a + 5% enriched tyrosine diet for 28 days compared to untreated control animals, respectively. Ion distribution maps of tyrosine (m/z 180.067) within tissue sections from control and treated (1 ppm and 10 ppm BCS-CR75391 respectively) animals of the thyroid gland after 14 days of treatment **a** and after 28 days of treatment **b** show enhanced tyrosine levels compared to the control. Intensity boxplots visualizing the intensity of the ion signal in every pixel (one dot ≙ one pixel) (c—d) confirm a significant enhancement of tyrosine levels in thyroid glands of animals treated with the HPPDi or tyrosine enriched diet. The significance test performed (t-test) shows a significant change among the individual groups (*** *p* < 0.001, ** *p* ≤ 0.002)

After 14 days exposure of rats to BCS-CR75391, the thyroid gland was removed. Thin sections were stained by hematoxylin and eosin to select sections suitable for mass spectrometry imaging analyses. A significant accumulation of free tyrosine in the thyroid could be detected by mass spectrometry imaging on a semi-quantitative level (Fig. 3a) in both dosage groups of 1 ppm and 10 ppm, in all the analyzed samples. The controls show no or only minimal tyrosine signals. These findings confirm the accumulation of tyrosine in the examined organs, which had been determined using UHPLC-MS.

The tyrosine within the thyroid gland tissue sections after 14 days both within 1 ppm and 10 ppm treatment group shows uniform and clear accumulation in comparison to the controls, with the strongest impact of treatment with 10 ppm (Fig. 3a, c). After 28 days and treatment with 2 ppm of HPPDi, however, the accumulation seems to be more spatially focused (Fig. 3b). A significant increase of tyrosine levels in comparison to the controls within these tissues could be proved with statistical significance test (*p* < 0.001). Both the imaging results of tyrosine distribution, but also the increased dispersion of the data points in the intensity plots (Fig. 3b, d) of the samples treated with 2 ppm of HPPDi BCS-CR75391 compared to the + 5% tyrosine diet and the controls indicate local accumulations of this amino acid within thyroid tissues. After 28 days of 2 ppm treatment, morphological changes in the form of visible lesions can be observed in histopathological analysis (Supplement Fig. 2). The initial assumption was that the tyrosine may accumulate within the colloid and could be responsible for the development of the lesions. The presented results, however, suggest that the tyrosine in the thyroid glands accumulates in the follicular epithelium and not in the colloid (Supplement Fig. 6).

### Accumulation of iodide in the thyroid

**Fig. 4:**
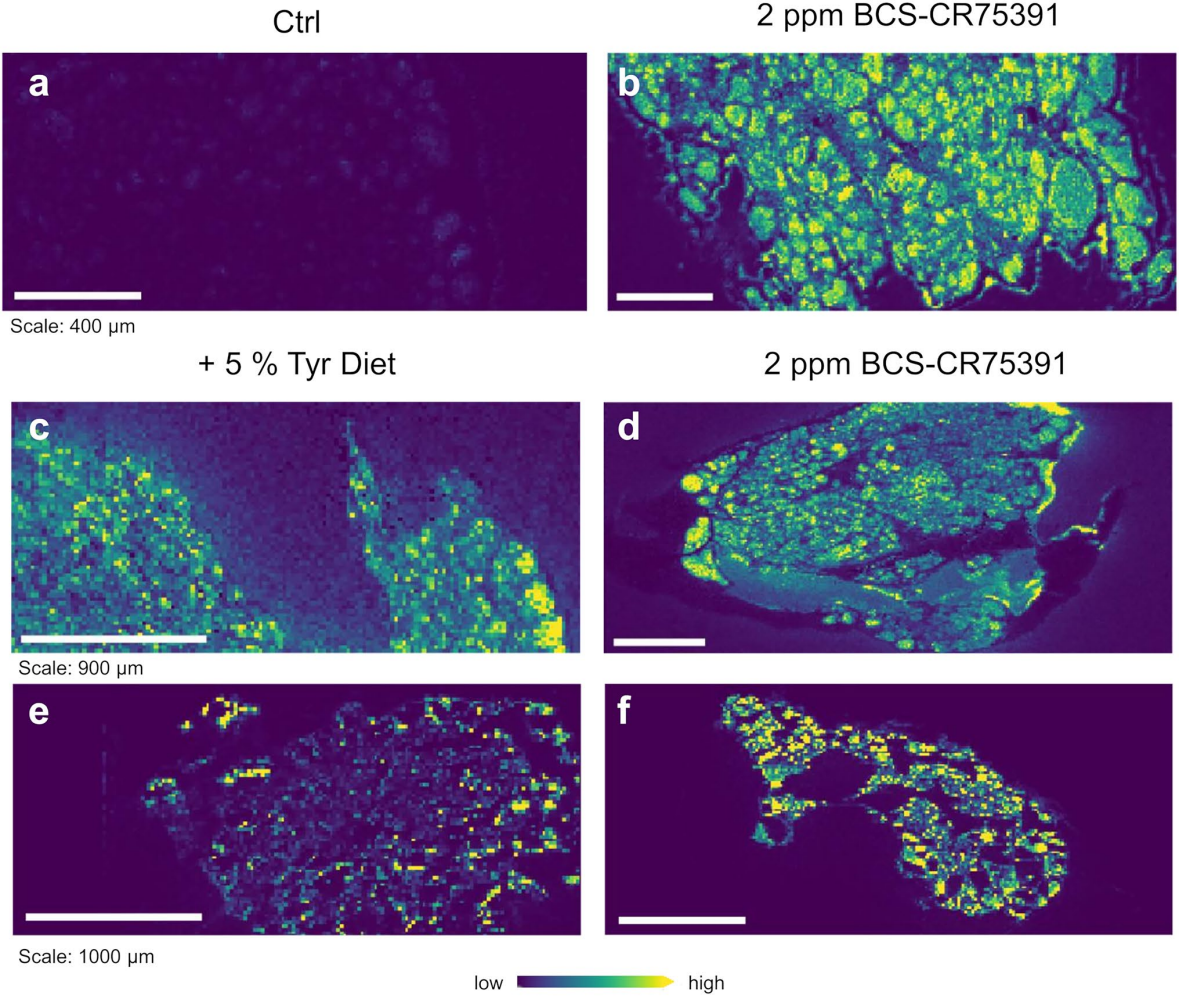
MALDI-MSI shows an accumulation of iodide (^127^I^−^, m/z 126.90) within the thyroid gland upon treatment of animals with 2 ppm HPPDi for 28 days. MALDI-MSI shows the localization of iodide within the colloid of rat thyroid glands. Moreover, the diet with 5% tyrosine also leads to an increase in iodide content in (c + e) but the pattern is less prominent and sparsely localized in comparison to the treatment with 2 ppm HPPDi (d + f)

By means of the MALDI-Orbitrap-MSI setup used in the present study, it was possible to visualize the two most important structural elements of the thyroid gland, the follicular cells and the colloid. Specific ion signals corresponding to the peak at m/z = 126.90 were detected, which correspond to the round to oval colloid (Fig. 4, shown in green and yellow), in which the thyroid hormones and precursors bound to thyroglobulin are stored, as well as regions in which thyrocytes are localized. Most interestingly, this colloid-specific signal represents the spatial localization of iodide, which is an essential element of the thyroid hormones. Treatment with HPPDi leads to a significant accumulation of iodide in the thyroid follicles (in all 10 examined sample sets). The results concerning the thyroid glands of rats treated with the + 5% tyrosine diet are not as clear as those of the animals treated with HPPDi. MALDI-Orbitrap-MS imaging has a limited spatial resolution of about 20 µm.

**Fig. 5:**
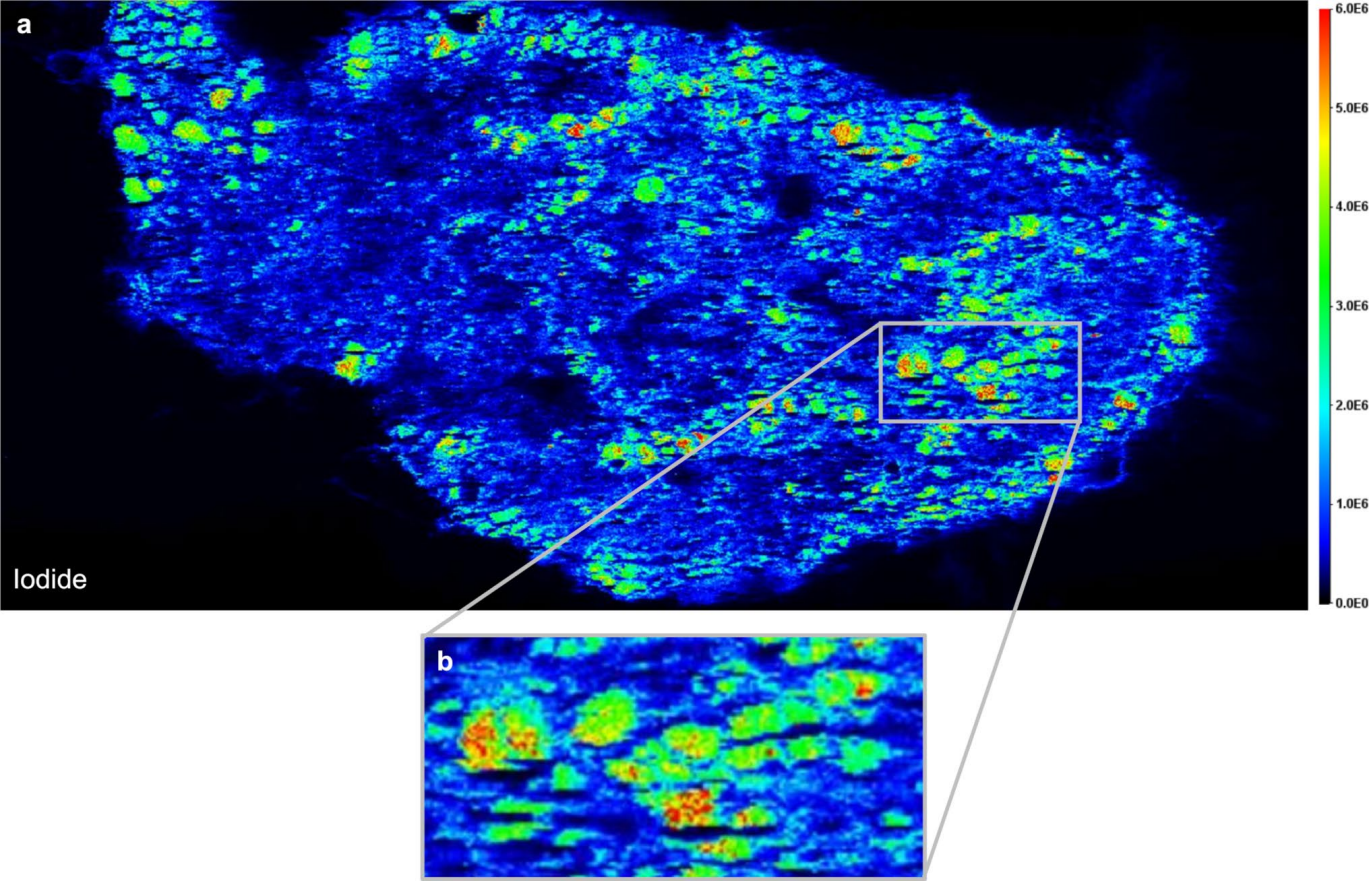
Distribution map of total iodide content within the thyroid gland of an HPPDi-treated rat measured by LA-ICP-MS. Localization of iodide in total within a thyroid gland tissue section of a rat treated with 2 ppm BCS-CR75391 over a period of 28 days was visualized via LA-ICP-MS, and it confirmed the observed iodide accumulation within the colloid of thyroid gland follicles **a**. Furthermore, a non-uniform distribution of iodide in the follicles can be proven with distinct hot spots within the colloid **b**

**Fig. 6:**
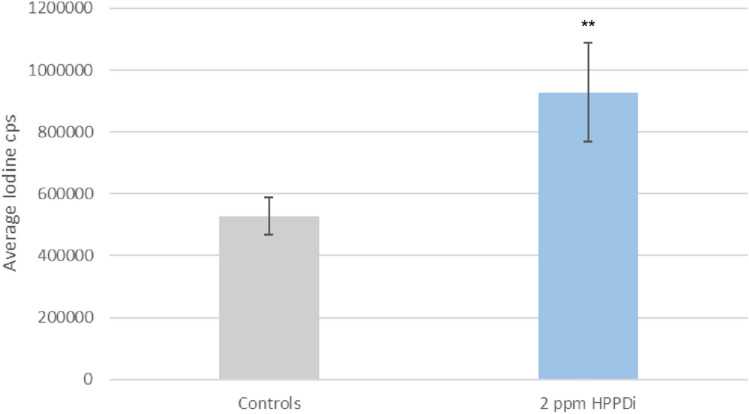
Relative quantification of the iodide signal within tissue sections of the thyroid gland of HPPDi (BCS-CR75391) treated (Treatment Period: 28 days) and control rats by LA-ICP-MS. A two-sided t-test confirms the significance of iodide accumulation within the tissue samples from HPPDi treated animals (** *p* < 0.05)

LA-ICP-MS was used to confirm the identification of iodide and obtain an even better spatial resolution. The identification of iodide as the localization within the follicular structures was confirmed by this approach (Fig. 5a). The spatial resolution of LA-ICP-MS also confirmed the non-even distribution of iodide within the follicles (Fig. 5b). Relative quantification reveals approximately 40% higher iodide levels in thyroid sections of animals treated with 2 ppm HPPDi compared to control animals (Fig. 6).

## Discussion

Treatment of rats with 1 ppm or 10 ppm BCS-CR75391 resulted in a significant increase of tyrosine levels in the blood. The tyrosine concentrations reached a plateau after 7 days of treatment, indicating a steady-state equilibrium between accumulation of tyrosine and its metabolic turnover and secretion. The concentrations observed in this study are in good correlation with other studies in rats (Lock et al. 1996; van Ravenzwaay et al. 2007; Antonenko et al. 2015). Tyrosine concentrations within all tissues analyzed, namely liver, pancreas, kidneys, eye, and thyroid gland, were higher than in the control tissues and the matching blood samples. These findings are also in good correlation with the effects reported in the rat in published literature (Keller et al. 2019; Botham et al. 2023), indicating that BCS-CR75391 exhibited a typical HPPD inhibitor response in this species. In summary, BCS-CR75391 exhibited the characteristic effect of HPPD inhibitors in rats on all levels analyzed.

Metabolome studies are well established to analyze toxicological effects of biologically active substances in cell cultures, test animals, and humans. In all tissues analyzed here, a characteristic, dosage-dependent shift of the metabolic patterns was observed. As expected, tyrosine was the leading metabolite that showed high abundances in all tissues. In a comparable study, van Ravenzwaay et al. (2007) analyzed the plasma of rats treated with HPPDi for metabolite profiles. This study also proved a moderate downregulation of a few amino acids and metabolites of the TCA cycle. The TCA cycle could be disturbed by a general disturbance of the cells; even so, the molecular reason remains unsolved. Multivariant statistics (PCA) showed a very clear separation of kidney and eye metabolite profiles from the controls, the 1 ppm and 10 ppm BCS-CR75391 treatments at 14 days. Liver and pancreas samples showed a clear separation of metabolite profiles for all treated versus non-treated animals, but the difference between dosages was not pronounced, indicating that even 1 ppm BCS-CR75391 exhibited an almost saturated effect. After 28 days of treatment, the clear separation of metabolite profiles for liver and pancreas was not observed anymore, while eye and kidney profiles were still separated. It seems that liver and pancreas metabolism adapted, and the profiles were similar to those of the control animals during this time of treatment; even so, the tyrosine levels remained high. This might also explain why the eye is more sensitive to toxic effects of HPPD inhibitors in rats, as shown in numerous studies (Lock et al. 2006).

The physiology and anatomy of the kidney make this organ especially susceptible to toxicologically relevant drug response (Griffin et al. 2019).

A significant accumulation of tyrosine was detected in all tissues analyzed. Special attention was given to the thyroid gland. Tyrosine seems to accumulate more in the follicular epithelium rather than in the colloid. An accumulation of iodide was observed within the thyroid gland following dietary administration of BCS-CR75391 to rats. Although iodide was annotated with high confidence by accurate mass and specific isotopic pattern by MALDI-Orbitrap-MSI, LA-ICP-MS provided an independent confirmation of these results. In addition, a higher spatial resolution could be obtained. Both approaches showed the accumulation of iodide within the follicles of thyroid glands of HPPDi treated animals. The better spatial resolution of LA-ICP-MS confirmed also the non-even distribution of iodide within the follicles. Mass spectrometry cannot distinguish between iodine (I_2_) and iodide (I^−^) since the ionization process of MALDI-MS, and even more of LA-ICP-MS, will convert iodine to iodide. In addition, the authors cannot rule out that organically bound iodine is released in the form of iodide by the MALDI process as in-source decay. In mass spectrometry, in-source decay refers to the fragmentation of ions directly in the source area of the mass spectrometer before they enter the analyzer. It is therefore possible that iodide is cleaved from thyroglobulin, which makes it challenging to precisely distinguish whether the accumulation is of free or bound iodide.

Former studies analyzing the effect of HPPDi in animals used histopathology and physiology to characterize the effects induced by tyrosinemia in the rat, the most sensitive species (Lock et al. 2006; Botham et al. 2023). The use of mass spectrometry imaging, as shown in this study, opens the possibility to bridge both fields, e.g., histopathology with quantification of the molecules of interest. The significant accumulation of tyrosine and iodine in thyroid glands of rats could be shown by MSI in a semi-quantitative way. This type of examination could also have been performed using laser microdissection and classical MS, which would have required a significant amount of additional effort and would also have resulted in the loss of further spatially resolved information recorded at the same time. Moreover, the analysis is performed on a single tissue section; classical analysis would require a higher amount of tissue samples. In conclusion, this study shows the important and innovative role of MALDI methodology in monitoring the accumulation of tyrosine induced by HPPDi in the target organs of rats.

The results of metabolic profiling, combined with untargeted, multivariant analysis, prove that lower concentrations of the substance, along with earlier detection of effects, may be sufficient for the detection of overall impact on multiple organs in toxicological studies with HPPDi in the rats. This could result in avoidance of high-concentrated treatments in animal studies and restrict these for the analysis of recovery effects or of long-termed exposures in accordance with the three Rs principle: replacement, reduction, refinement (Lauwereyns et al. 2024).

## Supplementary Information

Below is the link to the electronic supplementary material. 

Supplementary file1 (PDF 3003 KB)

## Data Availability

The data will be available on request.
