# Supplementary material for: Use of matrix-assisted laser desorption/ionization mass spectrometry imaging (MALDI-MSI) to visualize and support interpretation of toxic effects of 4-hydroxyphenylpyruvate dioxygenase inhibitors in rat tissues
Source: Arch Toxicol. 2025 Sep 30;100(1):247–58. doi: 10.1007/s00204-025-04175-0 (PMC12858625; doi:10.1007/s00204-025-04175-0)
Supplement: Supplementary file 1 — Supplementary file1 (PDF 3003 KB) [file 204_2025_4175_MOESM1_ESM.pdf]

Supplementary Information

Supplement Tab. 1 HPPDi (BCS-CR75391) concentrations within tissue samples of the liver, kidney and the pancreas of 14-day treated rats.
Quantification of the HPPDi (BCS-CR75391) used for the treatment before examination of toxicological effects within tissue samples of rats by UHPLC-MS. LOD = Limit of Detection, LOQ = Limit of Quantification, NA = Not Analyzed

14-Days Treatment with BCS-CR75391		
Liver	Ctrl. (n = 2)	< LOD
	1 ppm (n = 2)	0,085 ± 0,0003 nmol/mg
	10 ppm (n = 3)	0,089 ± 0,0027 nmol/mg
Kidney	Ctrl. (n = 0)	NA
	1 ppm (n = 1)	< LOQ
	10 ppm (n = 3)	< LOQ
Pancreas	Ctrl. (n = 0)	NA
	1 ppm (n = 0)	NA
	10 ppm (n = 3)	< LOD
LOD	0,058 nmol/mg	
LOQ	0,061 nmol/mg	

Supplement Tab. 2 HPPDi (BCS-CR75391) concentrations within tissue samples of the liver, kidney, pancreas and the eyes of 28-day treated rats.
Quantification of the HPPDi (BCS-CR75391) used for the treatment before examination of toxicological effects within tissue samples of rats by UHPLC-MS. LOD = Limit of Detection, LOQ = Limit of Quantification, NA = Not Analyzed

28-Days Treatment with BCS-CR75391		
Liver	Ctrl. (n = 3)	< LOD
	2 ppm (n = 3)	0,090 ± 0,0018 nmol/mg
Kidney	Ctrl. (n = 0)	NA
	2 ppm (n = 3)	< LOQ
Pancreas	Ctrl. (n = 0)	NA
	2 ppm (n = 3)	< LOD
Eye	Ctrl. (n = 0)	NA
	2 ppm (n = 3)	< LOD
LOD	0,058 nmol/mg	
LOQ	0,061 nmol/mg	

Schneider R.[1], Giampà M.[1], Schröder M. C.[1], Kubicki M.[2], Boyken J.[4], Beuret L.[3], Semino-Beninel G.[3], Niehaus K.[1], Schorsch F.[3], Lamshoeft M.[2], Bednarz H.[5]

(e-mail of the corresponding author: hanna.bednarz@uni-bielefeld.de)

Supplement Tab. 3 Main metabolites responsible for component separation in Principal Component Analysis (PCA).

This table presents the major metabolites contributing to the significant separation of components identified by PCA. Metabolites are listed by descending influence to indicate their importance in characterizing underlying biological or chemical differences

	14-Days	28-Days
Liver	Tyrosine	Tyrosine
	HPAA	Phenylalanine
	Fumarate	HPLA
	HPLA	
	Malate	
	Urea	
Pancreas	Tyrosine	Tyrosine
	HPLA	HPLA
	Fumarate	
	Malate	
	Urea	
	Glucose	
	Ethanolamine	
	HPAA	
Eyes	Tyrosine	Phenylalanine
	HPLA	HPLA
	HPAA	Tyrosine
	Glutamate	Fructose
	Phenylalanine	
	Fructose	
	Ethanolamine	
	Malate	
Kidneys	Tyrosine	Tyrosine
	Glutamate	HPLA
	HPLA	HPAA
	HPAA	Pantothenic Acid
	Phenylalanine	Glucose
	Glucose	Glutamate

Schneider R.[1], Giampà M.[1], Schröder M. C.[1], Kubicki M.[2], Boyken J.[4], Beuret L.[3], Semino-Beninel G.[3], Niehaus K.[1], Schorsch F.[3], Lamshoeft M.[2], Bednarz H.[5]

(e-mail of the corresponding author: hanna.bednarz@uni-bielefeld.de)

Supplement Tab. 4 Measurement parameters of targeted MALDI-Orbitrap-MSI analyses of tissue sections of the 14 days study

Parameter	Setting
**Resolution**	140,000
**Injection Time**	350 ms
**Polarity**	negative
**Scan Range**	50 to 250 m/z
**Lock Mass**	157.07712 m/z (DAN [M-H])
**Laser Frequency**	500 Hz
**Ion Funnel RF Frequency**	800.000 Hz
**Ion Funnel RF Drive**	15 %
**High Pressure Funnel RF Frequency**	596.000 Hz
**High Pressure Funnel RF Drive**	15 %
**Laser Current / Laser Energy**	2,3 A / ~ 12 µJ
**Raster Step Size**	20 µm
**Velocity**	2 mm / s

Supplement Tab. 5 Measurement parameters of targeted MALDI-Orbitrap-MSI analyses of FF tissue sections of the 28 days study

Parameter	Setting
**Resolution**	140,000
**Injection Time**	250 ms
**Polarity**	negative
**Scan Range**	65 to 250 m/z
**Lock Mass**	157.07712 m/z (DAN [M-H])
**Laser Frequency**	500 Hz
**Ion Funnel RF Frequency**	800.000 Hz
**Ion Funnel RF Drive**	15 %
**High Pressure Funnel RF Frequency**	596.000 Hz
**High Pressure Funnel RF Drive**	15 %
**Laser Current / Laser Energy**	2,1 A / ~ 8 µJ
**Raster Step Size**	20 µm
**Velocity**	1,5 mm / s

Schneider R.[1], Giampà M.[1], Schröder M. C.[1], Kubicki M.[2], Boyken J.[4], Beuret L.[3], Semino-Beninel G.[3], Niehaus K.[1], Schorsch F.[3], Lamshoeft M.[2], Bednarz H.[5]

(e-mail of the corresponding author: hanna.bednarz@uni-bielefeld.de)

Supplement Tab. 6 Measurement parameters of MALDI-Orbitrap-MSI analyses of FFPE tissue sections of the 28 days study

Parameter	Setting
**Resolution**	140,000
**Injection Time**	250 ms
**Polarity**	negative
**Scan Range**	65 to 250 m/z
**Lock Mass**	257.06178 m/z (NEDC [M-H])
**Laser Frequency**	500 Hz
**Ion Funnel RF Frequency**	800.000 Hz
**Ion Funnel RF Drive**	15 %
**High Pressure Funnel RF Frequency**	596.000 Hz
**High Pressure Funnel RF Drive**	15 %
**Laser Current / Laser Energy**	2,1 A / ~ 8 µJ
**Raster Step Size**	20 µm / 10 µm
**Velocity**	1,5 mm / s

Schneider R.[1], Giampà M.[1], Schröder M. C.[1], Kubicki M.[2], Boyken J.[4], Beuret L.[3], Semino-Beninel G.[3], Niehaus K.[1], Schorsch F.[3], Lamshoeft M.[2], Bednarz H.[5]

(e-mail of the corresponding author: hanna.bednarz@uni-bielefeld.de)

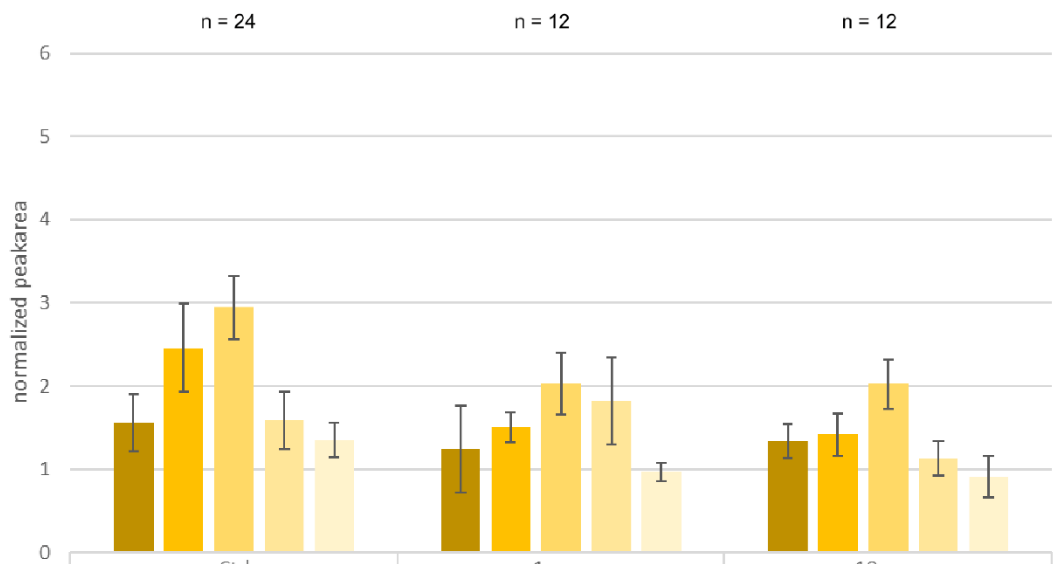

	Ctrl	1 ppm	10 ppm
Thyroid gland	1,553340318	1,24611	1,338862444
Pancreas	2,455967833	1,501877167	1,4164625
Liver	2,94325275	2,027176273	2,0242724
Eye	1,58513	1,816735556	1,135907778
Kidney	1,352054542	0,962975091	0,9121189

Supplement Fig. 1 Quantitation of glutamine within tissue samples of the thyroid gland, pancreas, eye, kidney and the liver of HPPDi (BCS-CR75391) treated and control rats.

Quantification of the metabolite glutamine by UHPLC-MS within tissue samples of the rats visualized as content bar garphs. HPPDi treatment for 14 days. A two-sided t-test confirms the significance of a slight decrease of glutamine content in the samples of the treated animals of both dose groups compared to the controls ($p < 0.01$) with exception of the thyroid gland. There was no significant difference in glutamine content between the two dose groups.

Schneider R.[1], Giampà M.[1], Schröder M. C.[1], Kubicki M.[2], Boyken J.[4], Beuret L.[3], Semino-Beninel G.[3], Niehaus K.[1], Schorsch F.[3], Lamshoeft M.[2], Bednarz H.[5]

(e-mail of the corresponding author: hanna.bednarz@uni-bielefeld.de)

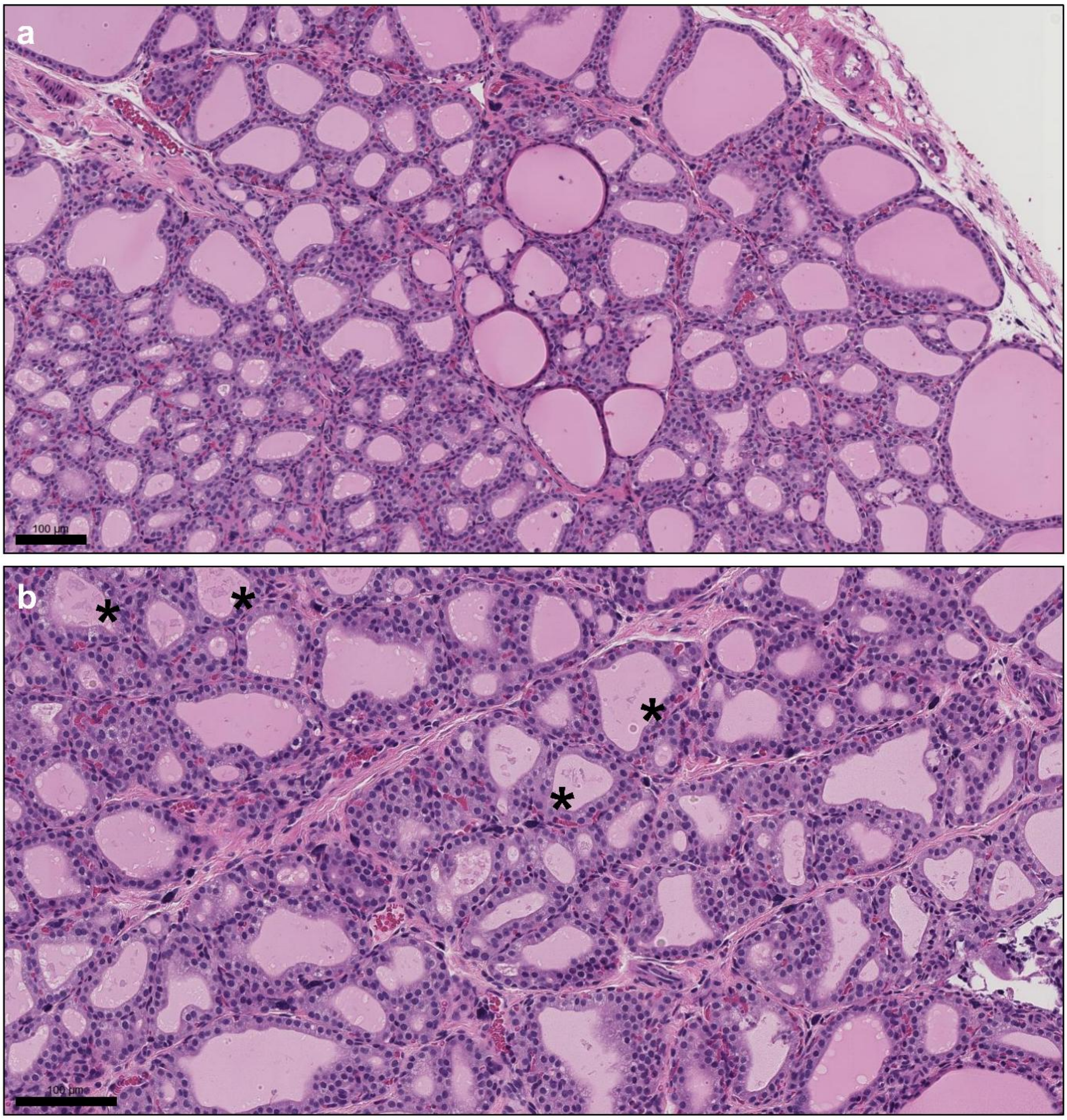

Supplement Fig. 2 Morphological comparison of tissue sections of the thyroid gland from control animals (a) and animals treated with an HPPDi for a period of 28 days (b).
Histological examination of thyroid tissue sections from control animals versus HPPDi-treated animals shows marked follicular cell hypertrophy and/or hyperplasia (FCHH). In addition, colloidal alterations (asterisks), which are typical for HPPDi treatment but do not occur in all cases, can be observed.

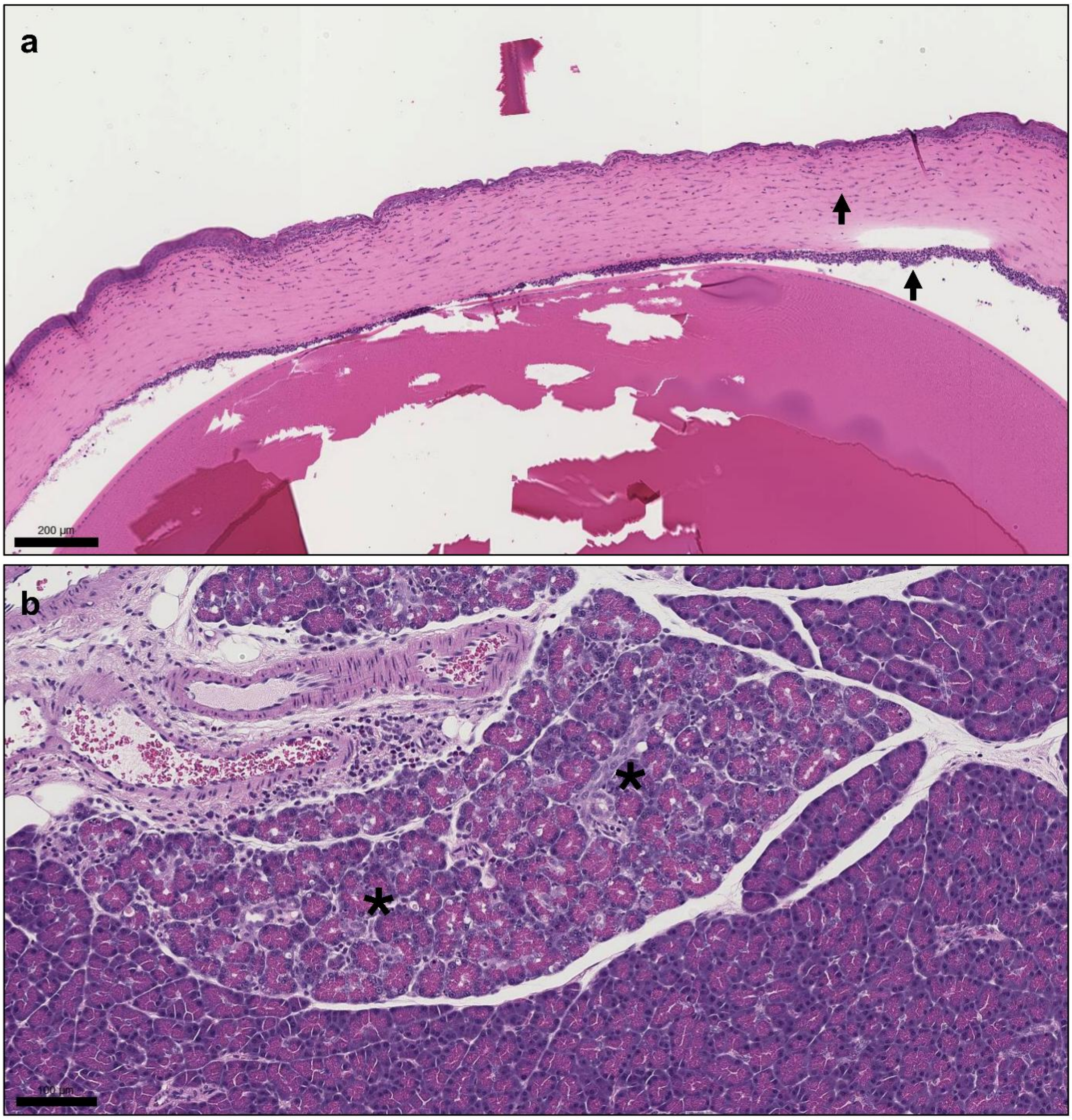

Supplement Fig. 3 Morphological appearance of typical lesions which can occur in the eye (a) and in the pancreas (b) during HPPDi treatment.
The rats in this case were treated over a period of 28 days with 2 ppm of the HPPDi BCS-CR75391. Typical lesions of the eye show a migration of immune cells (marked with arrows) to and into the cornea indicating corneal erosion and sometimes also diffuse keratitis. Cell atrophy and acinar degeneration are observed in the pancreas (marked with asterisks)

Schneider R.[1], Giampà M.[1], Schröder M. C.[1], Kubicki M.[2], Boyken J.[4], Beuret L.[3], Semino-Beninel G.[3], Niehaus K.[1], Schorsch F.[3], Lamshoeft M.[2], Bednarz H.[5]

Visualization and characterization of the toxicological effects of 4-hydroxyphenylpyruvate dioxygenase inhibitors in rodent tissues by matrix-assisted laser desorption/ionization mass spectrometry imaging
(e-mail of the corresponding author: hanna.bednarz@uni-bielefeld.de)

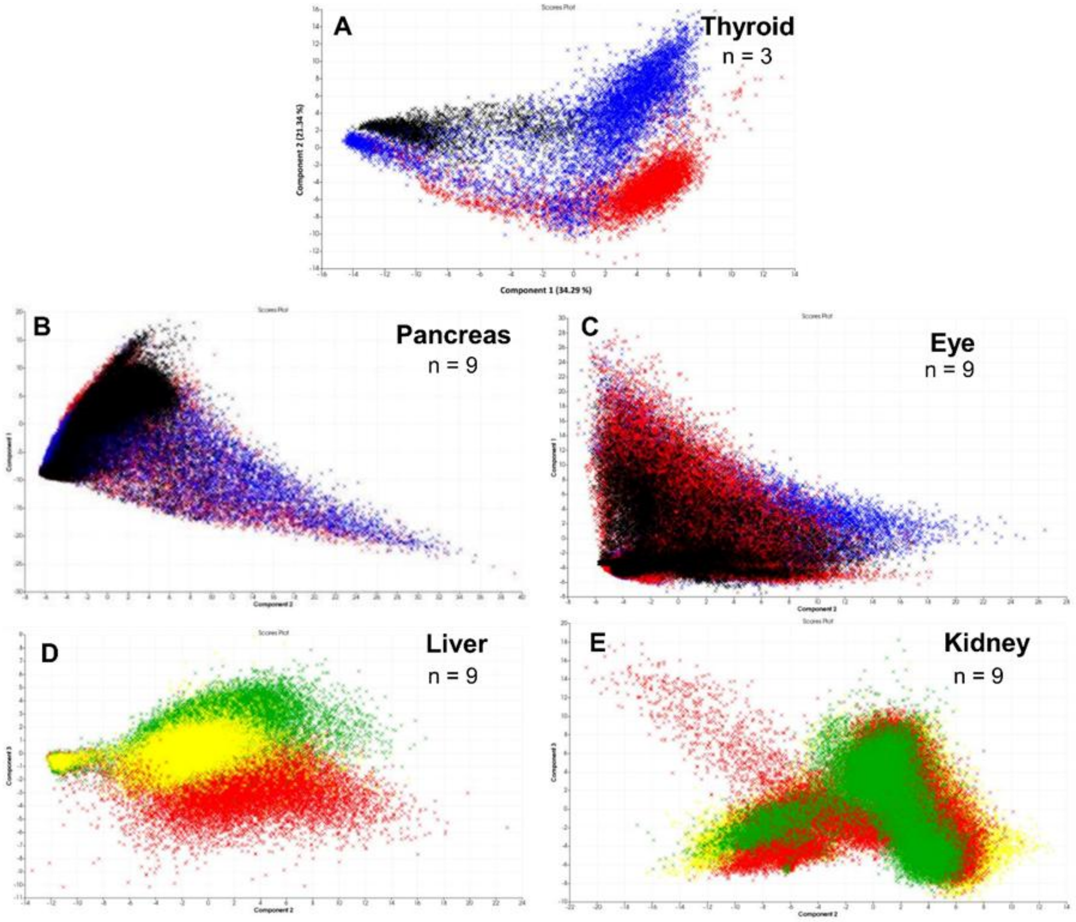

Supplement Fig. 4 Principal component analysis (PCA) of untargeted MALDI-MSI data of thyroid, pancreas, eye, liver and kidney tissue samples from rats treated with the HPPDi BCS-CR75391 for 14 days (1 ppm, 10 ppm) and untreated control animals.
Statistical analysis of untargeted MALDI-MSI data (matrix: DAN·HCl) of the organs of interest from male Wistar Rj: WI (IOPS HAN) after treatment with BCS-CR75391 and untreated by means of principal component analysis without prior knowledge concerning details of the samples. Black and green: Control, blue and yellow: 1 ppm, red: 10 ppm

Schneider R.[1], Giampà M.[1], Schröder M. C.[1], Kubicki M.[2], Boyken J.[4], Beuret L.[3], Semino-Beninel G.[3], Niehaus K.[1], Schorsch F.[3], Lamshoeft M.[2], Bednarz H.[5]

(e-mail of the corresponding author: hanna.bednarz@uni-bielefeld.de)

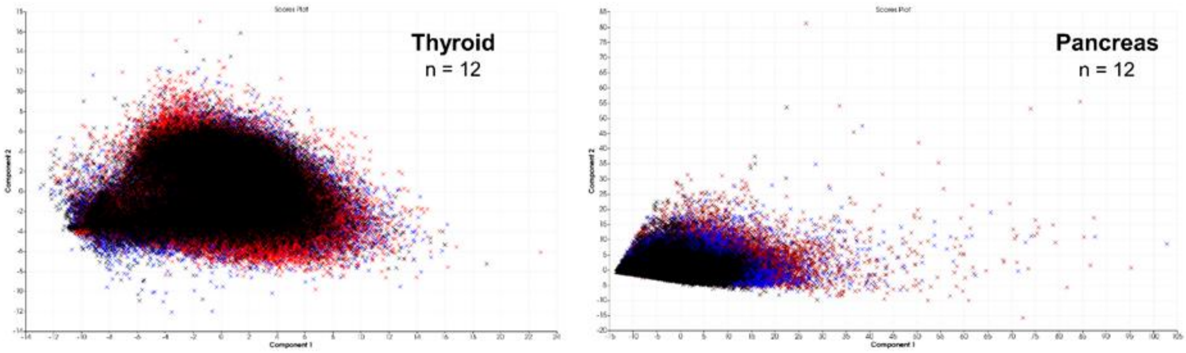

Supplement Fig. 5 Principal component analysis (PCA) of untargeted MALDI-MSI data of thyroid gland and pancreas tissue samples from rats treated with 2 ppm of the HPPDi BCS-CR75391 or received a + 5 % enriched tyrosine diet for 28 days and untreated control animals.
Statistical analysis of untargeted MALDI-MSI data (matrix: DAN·HCl) of the thyroid gland and pancreas from male Wistar Rj: WI (IOPS HAN) after treatment with BCS-CR75391, a + 5 % enriched tyrosine diet and untreated by means of principal component analysis without prior knowledge concerning details of the samples. Black: Control, blue: + 5 % tyrosine diet, red: 2 ppm

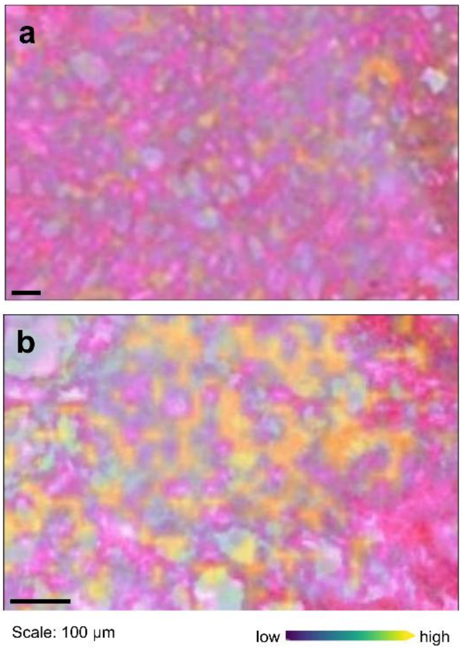

Supplement Fig. 6 Overlay of an ion distribution map showing the *in-situ* localization of free tyrosine within a thyroid gland tissue section after 14 days of exposition to 1 ppm (a) and 10 ppm (b) of BCS-CR75391.
The visualization of the free tyrosine distribution within the thyroid gland of an HPPDi treated rat via MALDI-MSI indicates a localization of the accumulated tyrosine within the thyrocytes und the surrounding tissue and not within the colloid as initially assumed

Schneider R.[1], Giampà M.[1], Schröder M. C.[1], Kubicki M.[2], Boyken J.[4], Beuret L.[3], Semino-Beninel G.[3], Niehaus K.[1], Schorsch F.[3], Lamshoeft M.[2], Bednarz H.[5]

(e-mail of the corresponding author: hanna.bednarz@uni-bielefeld.de)

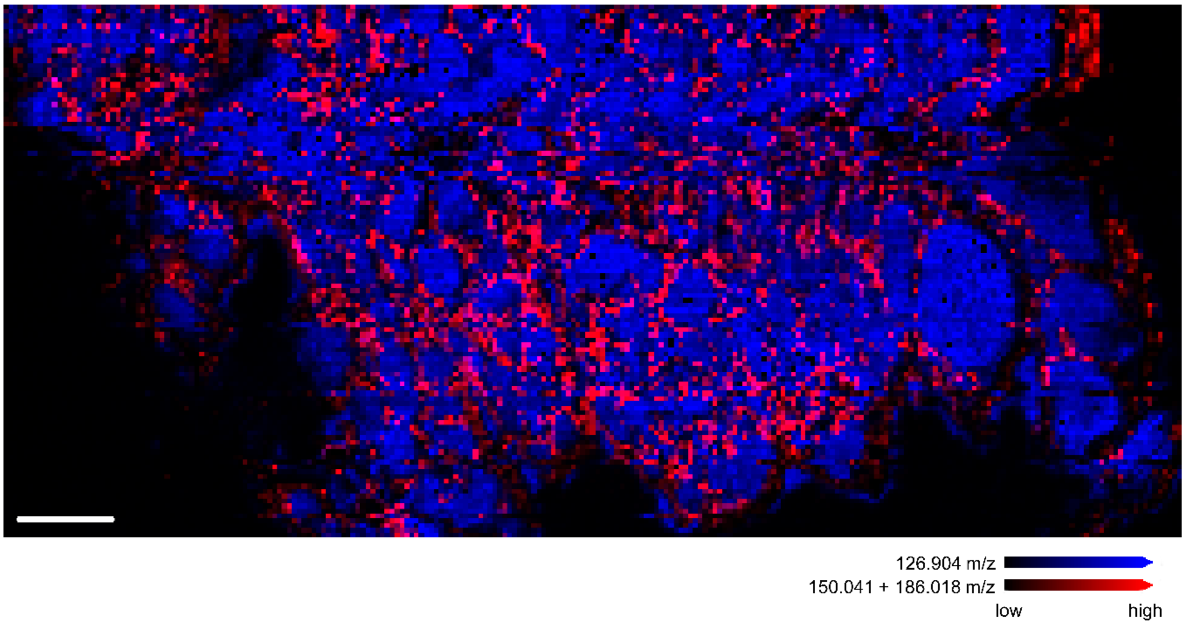

Supplement Fig. 7 Visualization of the main thyroid gland structures using MALDI-MSI.
Illustration of the most important structures of the thyroid gland for the present work, the follicular epithelium (thyrocytes) and the colloid, based on the ion distribution of the metabolites specific for the respective areas. The red signal corresponds to a non-identified metabolite specific for the thyrocytes, appearing as M-H- and M+Cl- ions. The blue signal corresponds to iodine that is localized in the colloid, Scale-Bar: 200 µm

Schneider R.[1], Giampà M.[1], Schröder M. C.[1], Kubicki M.[2], Boyken J.[4], Beuret L.[3], Semino-Beninel G.[3], Niehaus K.[1], Schorsch F.[3], Lamshoeft M.[2], Bednarz H.[5]

(e-mail of the corresponding author: hanna.bednarz@uni-bielefeld.de)

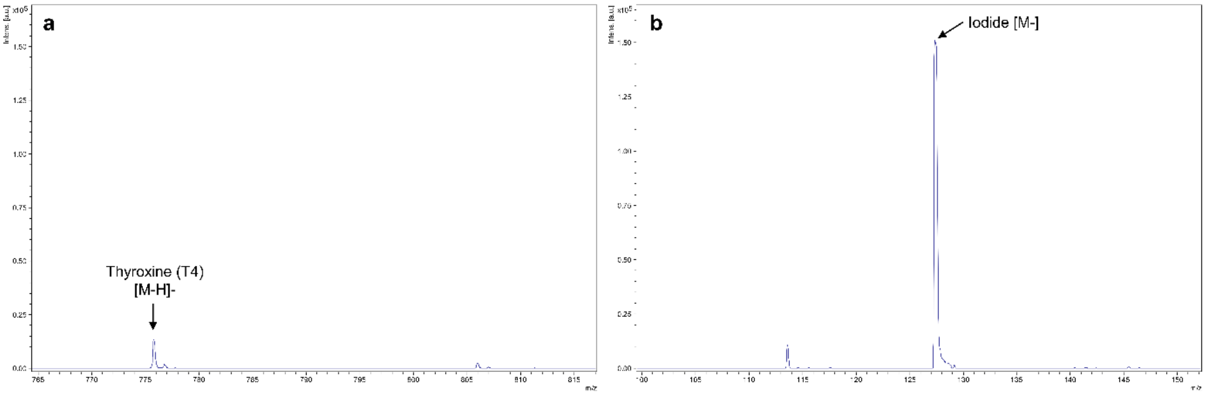

Supplement Fig. 8 MALDI-ToF-MS of pure T4 as reference using DAN as matrix in negative polarity. The measurement confirms the possible detection of T3 and T4 using MALDI-ToF-MS with DAN as MALDI matrix. The mass spectra show both, the signal $(M\text{-}H)^-$ of T4 (m/z 776) (a) and an iodide signal (m/z 127) most probably caused by a fragmentation of T4 (b)

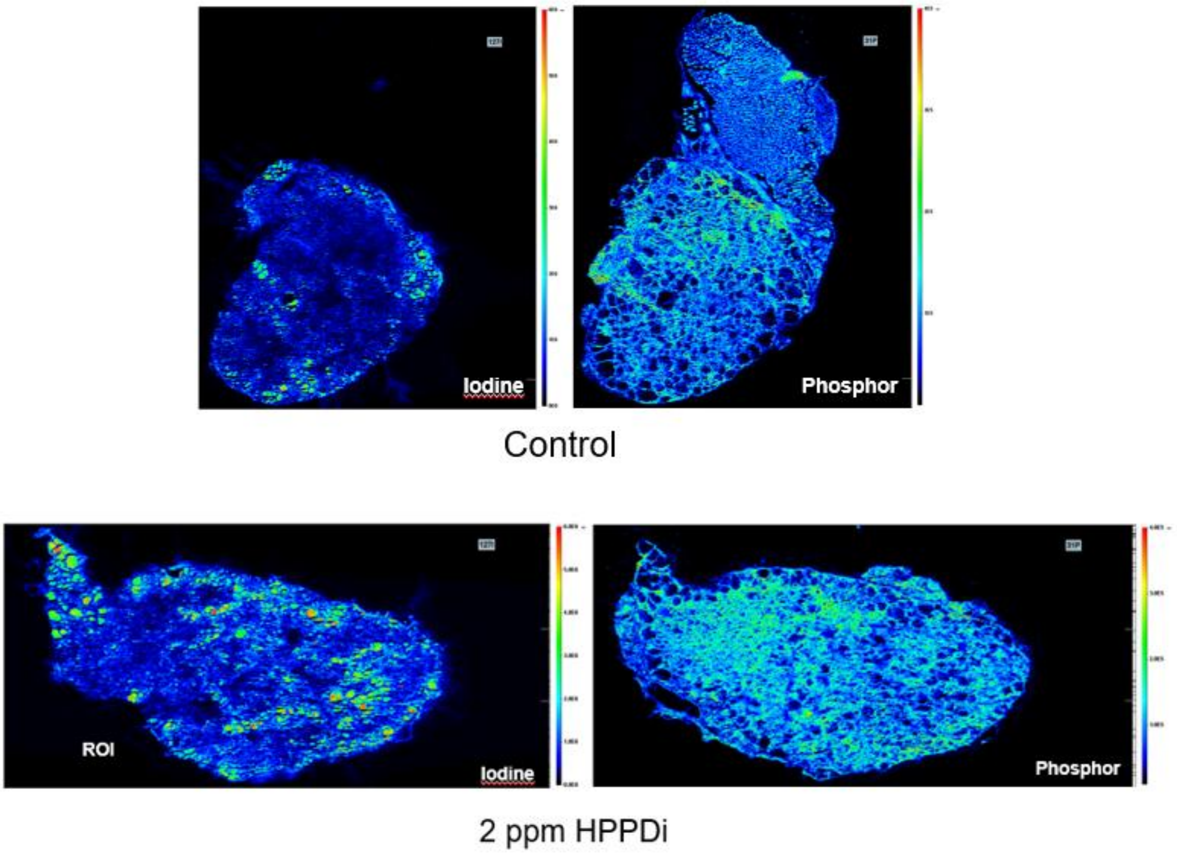

Supplement Fig. 9 LA-ICP-MSI confirms the accumulation of iodine within the thyroid gland upon treatment of animals with HPPDi observed using MALDI-MSI.

Schneider R.[1], Giampà M.[1], Schröder M. C.[1], Kubicki M.[2], Boyken J.[4], Beuret L.[3], Semino-Beninel G.[3], Niehaus K.[1], Schorsch F.[3], Lamshoeft M.[2], Bednarz H.[5]

(e-mail of the corresponding author: hanna.bednarz@uni-bielefeld.de)

LA-ICP-MSI shows the localization of iodine within the colloid of rat thyroid glands in comparison to the phosphorous signal. It can be observed that treatment with HPPDi leads to an associated increase in the iodine content in the colloid

Schneider R.[1], Giampà M.[1], Schröder M. C.[1], Kubicki M.[2], Boyken J.[4], Beuret L.[3], Semino-Beninel G.[3], Niehaus K.[1], Schorsch F.[3], Lamshoeft M.[2], Bednarz H.[5]

Visualization and characterization of the toxicological effects of 4-hydroxyphenylpyruvate dioxygenase inhibitors in rodent tissues by matrix-assisted laser desorption/ionization mass spectrometry imaging
(e-mail of the corresponding author: hanna.bednarz@uni-bielefeld.de)

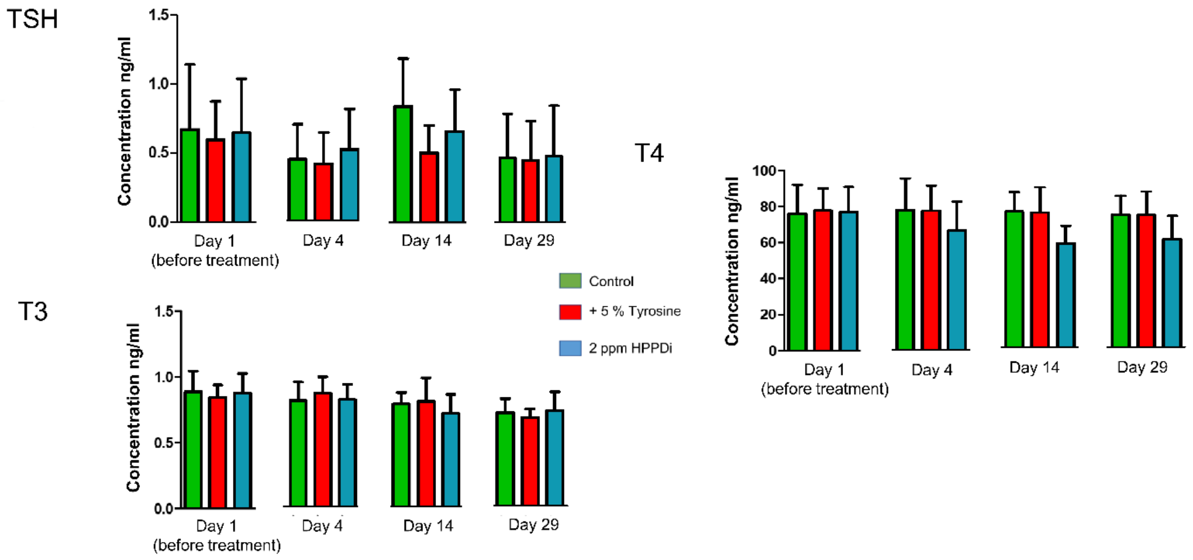

Supplement Fig. 10 Mean blood concentration of the thyroid hormones 3-iodothyronine (T3) and thyroxine (T4) and the thyroid stimulating hormone (TSH) at different time points during the HPPDi treatment.
Measurements of blood concentrations of T3, T4 and TSH by HPLC-MS show no alterations of blood levels as consequence of the HPPDi treatment

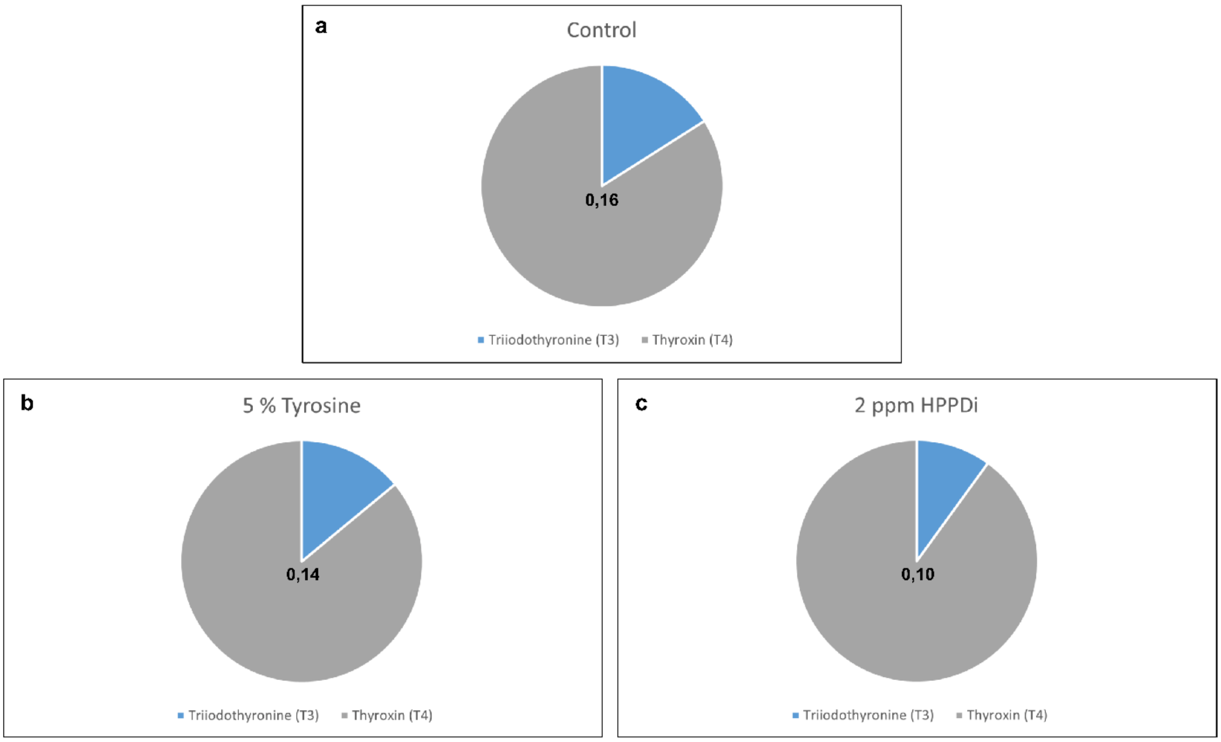

Supplement Fig. 11 Pie chart depicting the T3 to T4 thyroid gland hormone ratio, illustrating the distribution of the total hormone content within the thyroid gland tissue among each other.
T3 and T4 are shown as a percentage of the total hormone content. As shown in the chart legend, T3 is shown in gray and T4 in blue. In the center of each pie chart, the T3/T4 ratio is displayed as a number

Schneider R.[1], Giampà M.[1], Schröder M. C.[1], Kubicki M.[2], Boyken J.[4], Beuret L.[3], Semino-Beninel G.[3], Niehaus K.[1], Schorsch F.[3], Lamshoeft M.[2], Bednarz H.[5]

(e-mail of the corresponding author: hanna.bednarz@uni-bielefeld.de)

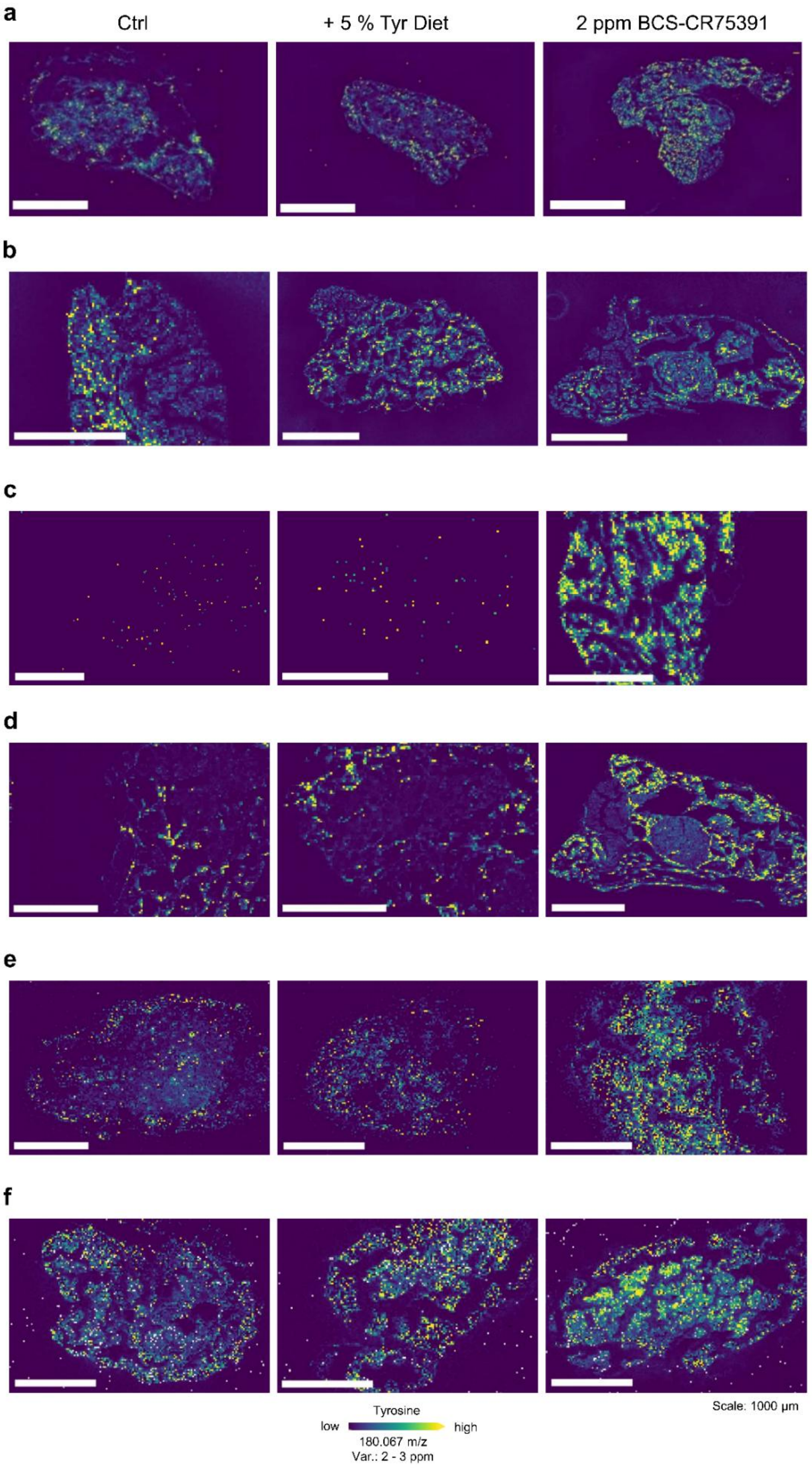

Schneider R.[1], Giampà M.[1], Schröder M. C.[1], Kubicki M.[2], Boyken J.[4], Beuret L.[3], Semino-Beninel G.[3], Niehaus K.[1], Schorsch F.[3], Lamshoeft M.[2], Bednarz H.[5]

Visualization and characterization of the toxicological effects of 4-hydroxyphenylpyruvate dioxygenase inhibitors in rodent tissues by matrix-assisted laser desorption/ionization mass spectrometry imaging
(e-mail of the corresponding author: hanna.bednarz@uni-bielefeld.de)

Archives of Toxicology

Supplement Fig. 12 Tyrosine distribution within the thyroid gland of rats treated with the HPPDi BCS-CR75391 for 28 days and with a to 5 % tyrosine enriched diet for 28 days compared to untreated control animals.
Ion distribution maps of tyrosine (m/z 180.067) visualized by MALDI-Orbitrap-MSI within tissue sections from control and treated rats after 28-days of treatment of six replicates show enhanced tyrosine levels compared to the control

Schneider R.[1], Giampà M.[1], Schröder M. C.[1], Kubicki M.[2], Boyken J.[4], Beuret L.[3], Semino-Beninel G.[3], Niehaus K.[1], Schorsch F.[3], Lamshoeft M.[2], Bednarz H.[5]

(e-mail of the corresponding author: hanna.bednarz@uni-bielefeld.de)

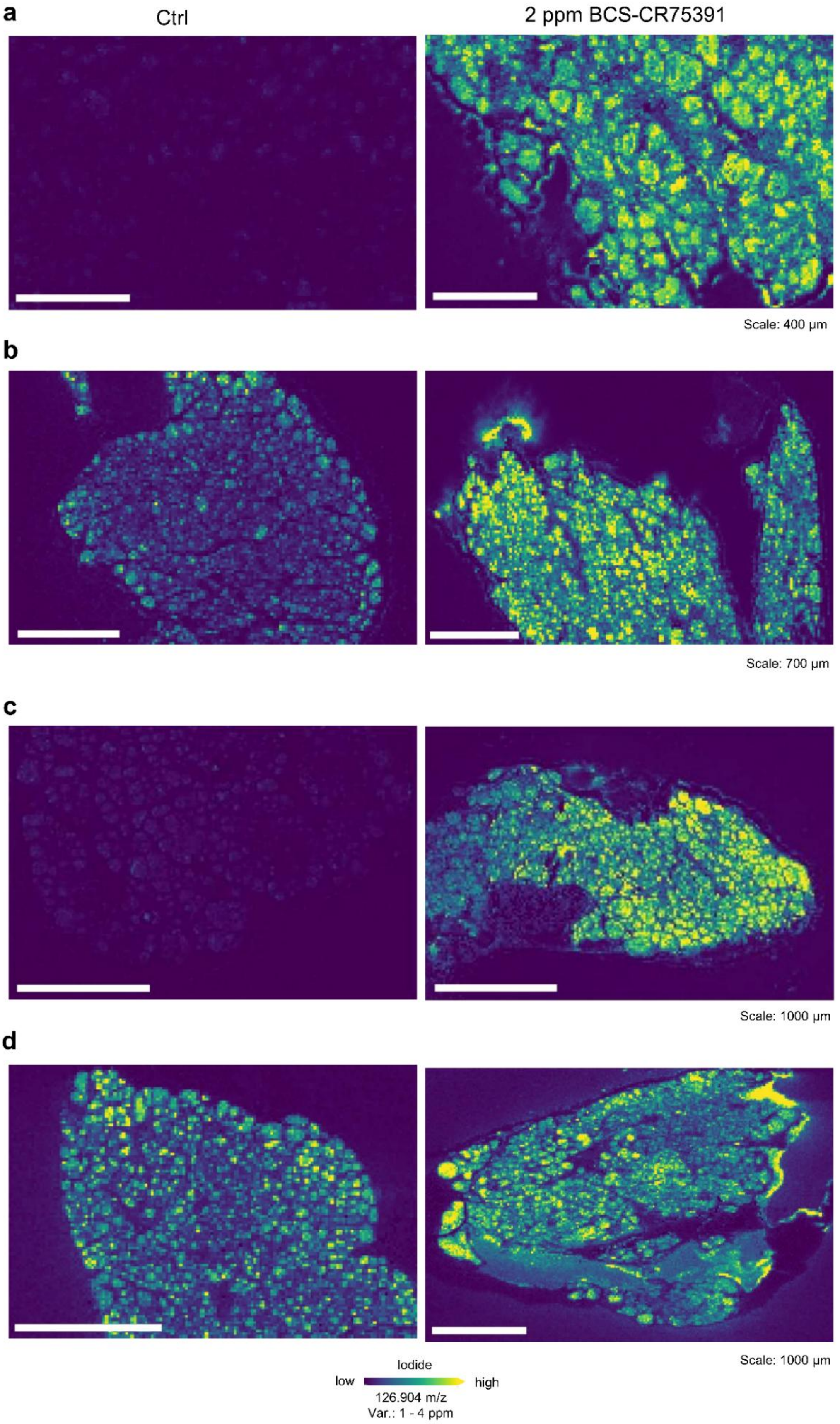

Schneider R.[1], Giampà M.[1], Schröder M. C.[1], Kubicki M.[2], Boyken J.[4], Beuret L.[3], Semino-Beninel G.[3], Niehaus K.[1], Schorsch F.[3], Lamshoeft M.[2], Bednarz H.[5]

Visualization and characterization of the toxicological effects of 4-hydroxyphenylpyruvate dioxygenase inhibitors in rodent tissues by matrix-assisted laser desorption/ionization mass spectrometry imaging

(e-mail of the corresponding author: hanna.bednarz@uni-bielefeld.de)

Archives of Toxicology

Supplement Fig. 13 Iodide distribution within the thyroid gland of rats treated with the HPPDi BCS-CR75391 for 28 days compared to untreated control animals.

Ion distribution maps of iodide (m/z 126.904) visualized by MALDI-Orbitrap-MSI within FFPE tissue sections from control and treated rats after 28-days of treatment of four replicates. The tissue sections of treated animals show enhanced iodide levels compared to the control sections

Schneider R.[1], Giampà M.[1], Schröder M. C.[1], Kubicki M.[2], Boyken J.[4], Beuret L.[3], Semino-Beninel G.[3], Niehaus K.[1], Schorsch F.[3], Lamshoeft M.[2], Bednarz H.[5]

(e-mail of the corresponding author: hanna.bednarz@uni-bielefeld.de)

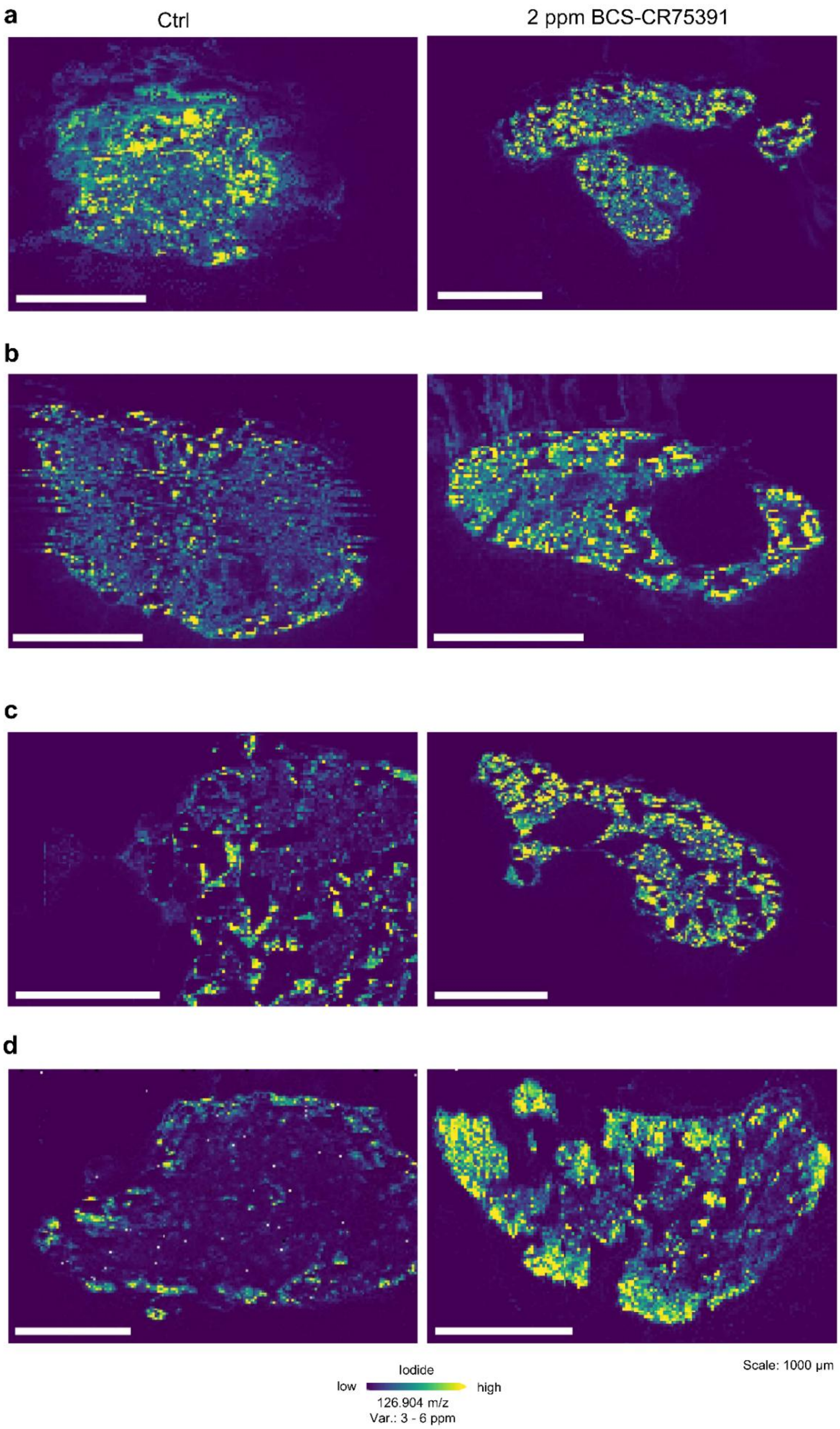

Schneider R.[1], Giampà M.[1], Schröder M. C.[1], Kubicki M.[2], Boyken J.[4], Beuret L.[3], Semino-Beninel G.[3], Niehaus K.[1], Schorsch F.[3], Lamshoeft M.[2], Bednarz H.[5]

Visualization and characterization of the toxicological effects of 4-hydroxyphenylpyruvate dioxygenase inhibitors in rodent tissues by matrix-assisted laser desorption/ionization mass spectrometry imaging

(e-mail of the corresponding author: hanna.bednarz@uni-bielefeld.de)

Archives of Toxicology

Supplement Fig. 14 Iodide distribution within the thyroid gland of rats treated with the HPPDi BCS-CR75391 for 28 days compared to untreated control animals.
Ion distribution maps of iodide (m/z 126.904) visualized by MALDI-Orbitrap-MSI within FF tissue sections from control and treated rats after 28-days of treatment of four replicates. The tissue sections of treated animals show enhanced iodide levels compared to the control sections

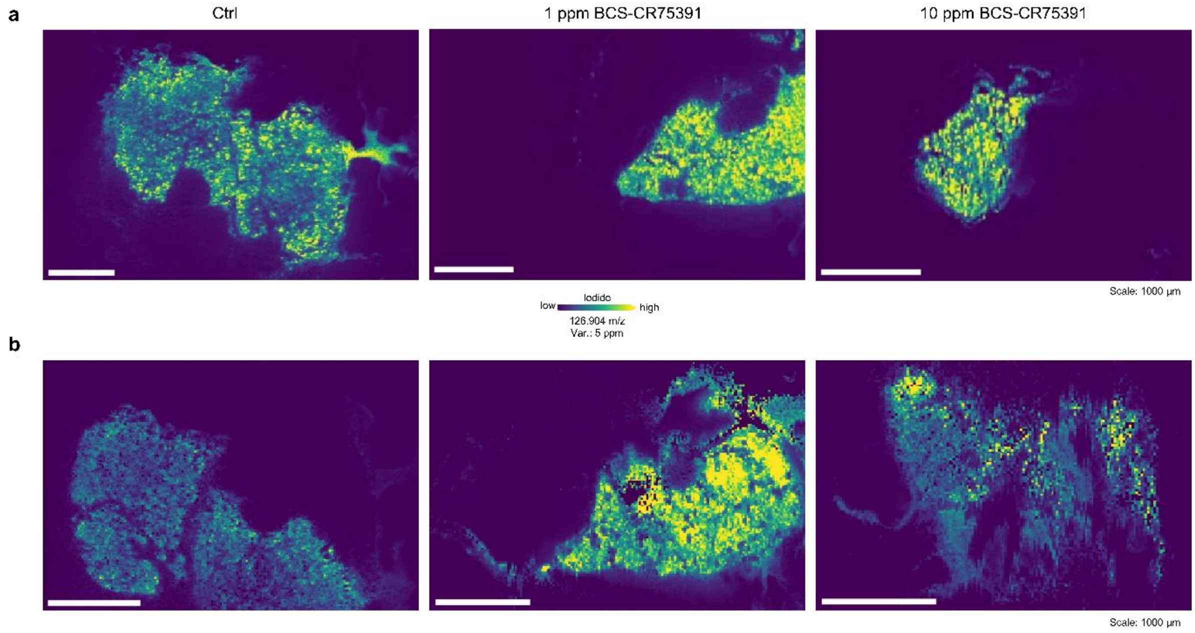

Supplement Fig. 15 Iodide distribution within the thyroid gland of rats treated with the HPPDi BCS-CR75391 for 14 days with a dose of 1 and 10 ppm compared to untreated control animals.
Ion distribution maps of iodide (m/z 126.904) visualized by MALDI-Orbitrap-MSI within FF tissue sections from control and treated rats after 14-days of treatment of two replicates. The tissue sections of treated animals show enhanced iodide levels compared to the control sections

Schneider R.[1], Giampà M.[1], Schröder M. C.[1], Kubicki M.[2], Boyken J.[4], Beuret L.[3], Semino-Beninel G.[3], Niehaus K.[1], Schorsch F.[3], Lamshoeft M.[2], Bednarz H.[5]

(e-mail of the corresponding author: hanna.bednarz@uni-bielefeld.de)

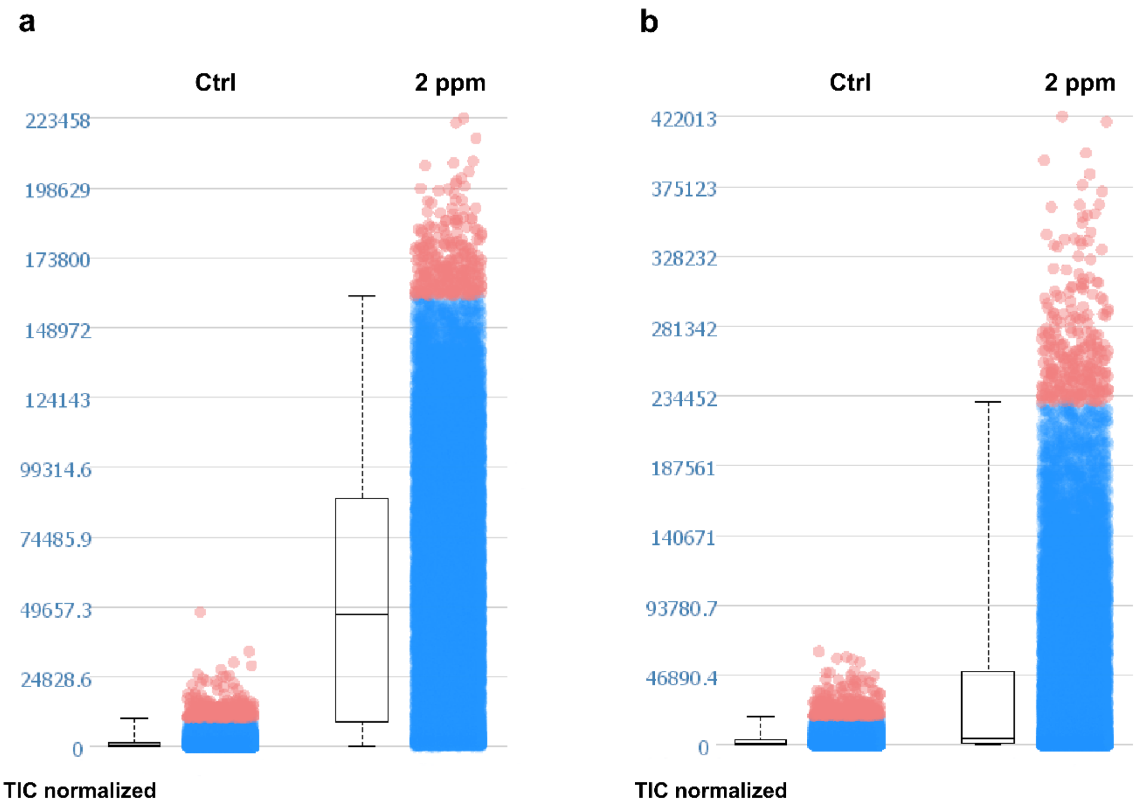

Supplement Fig. 16 Intensity boxplots of MALDI-MSI iodide (m/z 126.90) signal within the thyroid gland of animals treated with HPPDi compared to non-treated control animals.
Signal intensity of iodide confirms the accumulation of iodide within the thyroid glands of rats treated with HPPDi

Schneider R.[1], Giampà M.[1], Schröder M. C.[1], Kubicki M.[2], Boyken J.[4], Beuret L.[3], Semino-Beninel G.[3], Niehaus K.[1], Schorsch F.[3], Lamshoeft M.[2], Bednarz H.[5]

Visualization and characterization of the toxicological effects of 4-hydroxyphenylpyruvate dioxygenase inhibitors in rodent tissues by matrix-assisted laser desorption/ionization mass spectrometry imaging
(e-mail of the corresponding author: hanna.bednarz@uni-bielefeld.de)

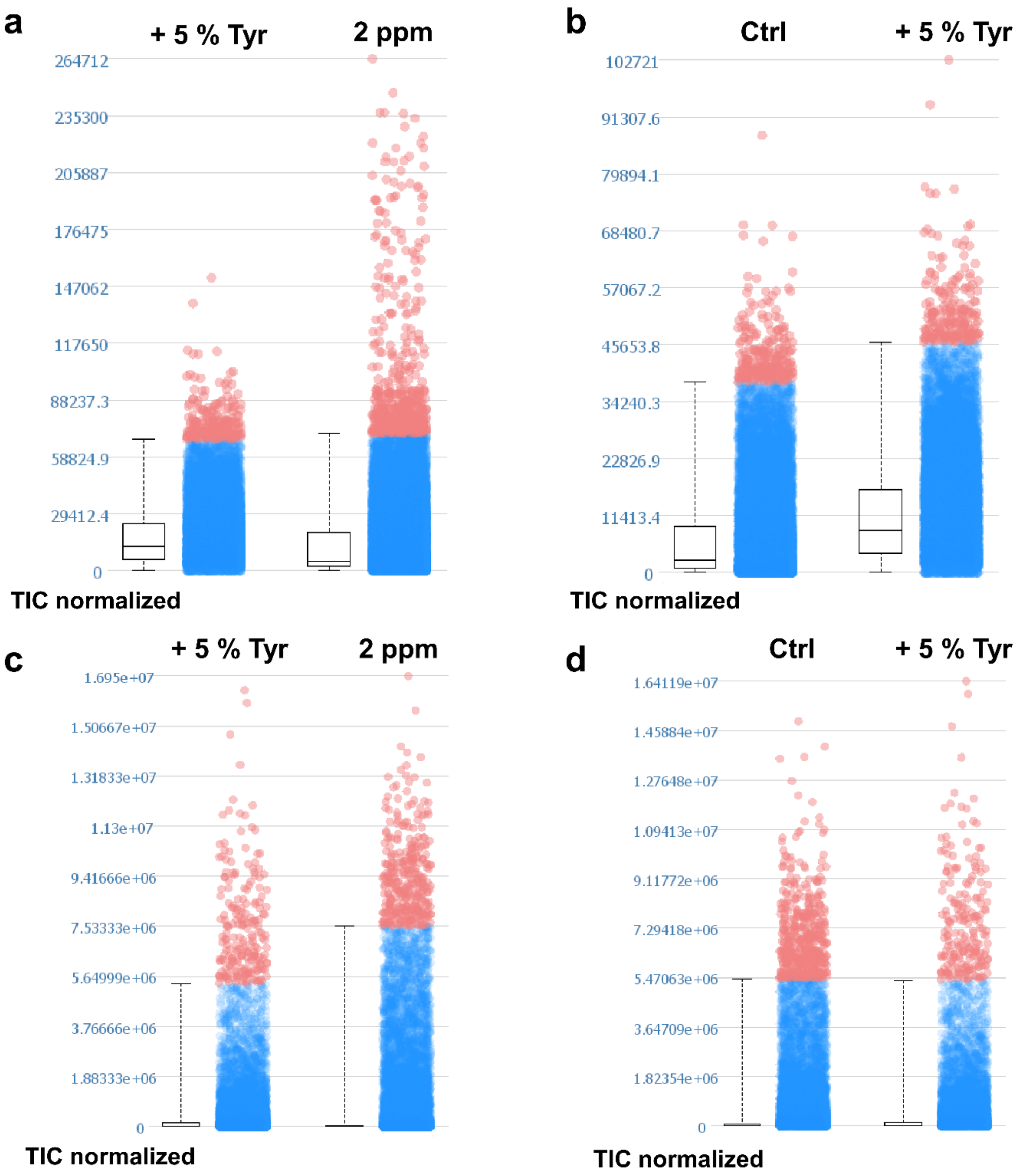

Supplement Fig. 17 Intensity boxplots of MALDI-MSI iodide (m/z 126.90) signal within the thyroid gland of animals treated with an excess of l-tyrosine compared to non- and HPPDi-treated animals.
Signal intensity of iodide elucidates the vicissitude of iodide accumulation within the thyroid glands of rats treated with HPPDi, excess of l-tyrosine and non-treated

Schneider R.[1], Giampà M.[1], Schröder M. C.[1], Kubicki M.[2], Boyken J.[4], Beuret L.[3], Semino-Beninel G.[3], Niehaus K.[1], Schorsch F.[3], Lamshoeft M.[2], Bednarz H.[5]

Visualization and characterization of the toxicological effects of 4-hydroxyphenylpyruvate dioxygenase inhibitors in rodent tissues by matrix-assisted laser desorption/ionization mass spectrometry imaging
(e-mail of the corresponding author: hanna.bednarz@uni-bielefeld.de)
